# Abyssal fauna of the UK-1 polymetallic nodule exploration claim, Clarion-Clipperton Zone, central Pacific Ocean: Echinodermata

**DOI:** 10.3897/BDJ.4.e7251

**Published:** 2016-01-25

**Authors:** Adrian G Glover, Helena Wiklund, Muriel Rabone, Diva J Amon, Craig R Smith, Tim O'Hara, Christopher L Mah, Thomas G Dahlgren

**Affiliations:** ‡ Natural History Museum, London, United Kingdom; § University of Hawaii, Honolulu, United States of America; | Museum Victoria, Melbourne, Australia; ¶ Smithsonian Institution National Museum of Natural History, Washington, United States of America; # Uni Research, Bergen, Norway; ¤ University of Gothenburg, Dep. Marine Sciences, Gothenburg, Sweden

## Abstract

We present data from a DNA taxonomy register of the abyssal benthic Echinodermata collected as part of the Abyssal Baseline (ABYSSLINE) environmental survey cruise ‘AB01’ to the UK Seabed Resources Ltd (UKSRL) polymetallic-nodule exploration claim ‘UK-1’ in the eastern Clarion-Clipperton Zone (CCZ), central Pacific Ocean abyssal plain. Morphological and genetic data are presented for 17 species (4 Asteroidea, 4 Crinoidea, 2 Holothuroidea and 7 Ophiuroidea) identified by a combination of morphological and genetic data. No taxa matched previously published genetic sequences, but 8 taxa could be assigned to previously-described species based on morphology, although here we have used a precautionary approach in taxon assignments to avoid over-estimating species ranges. The Clarion-Clipperton Zone is a region undergoing intense exploration for potential deep-sea mineral extraction. We present these data to facilitate future taxonomic and environmental impact study by making both data and voucher materials available through curated and accessible biological collections.

## Introduction

We present data from a DNA taxonomy register of the abyssal benthic Echinodermata collected as part of the Abyssal Baseline (ABYSSLINE) environmental survey cruise ‘AB01’ to the UK Seabed Resources Ltd (UKSRL) polymetallic-nodule exploration claim ‘UK-1’ in the eastern Clarion-Clipperton Zone (CCZ), central Pacific Ocean (Smith et al. 2013).

This paper is the start of an iterative approach to providing regional taxonomic synthesis for a region that is undergoing intense deep-sea mineral exploration for high-grade polymetallic nodules regulated by Sponsoring States (here the United Kingdom Government) and the International Seabed Authority (ISA 2014b, Glover and Smith 2003, Wedding et al. 2015). Our study is not yet a comprehensive faunal guide to the region, but a data paper that will be updated with new additions following future collections and analyses. New versions will contain all the data contained in the previous version, plus additional descriptions and records from future research cruises.

The abyssal zone of the world’s oceans has been defined as the seafloor between depths of 3000m and 6000m, a bathymetric zone that encompasses 54% of the geographic surface of the planet (Smith et al. 2008). Echinoderms form a characteristic and abundant group in this region. Current online data sources list 698 echinoderm species recorded at abyssal depths from between 3000m and 6000m (OBIS 2015) out of a total of 3,272 echinoderm species recorded from depths greater than 500m (Glover et al. 2015).

**Figure 1.**
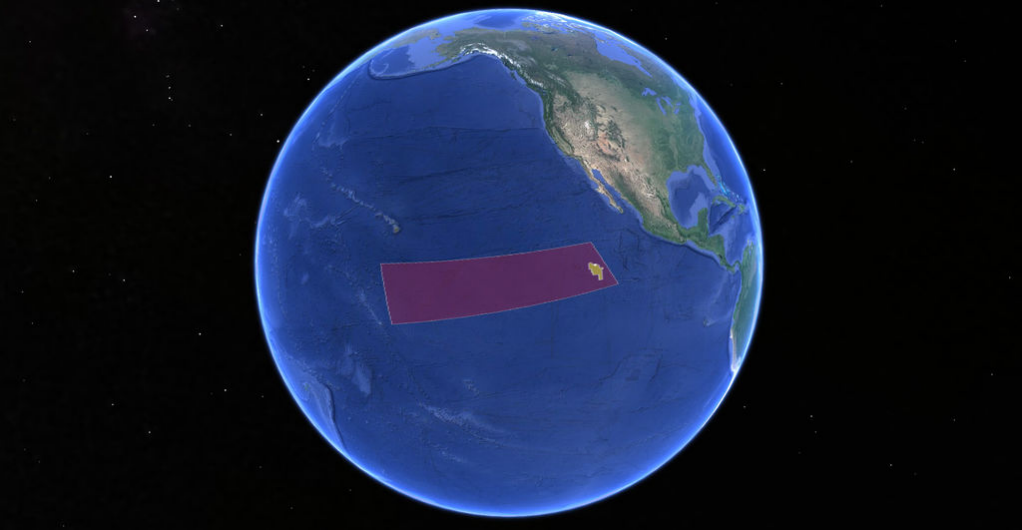
The Clarion-Clipperton Zone, central Pacific Ocean (purple box) is a 6 milllion km^2^ region at the time of writing containing only 290 online-databased records of echinoderm species (OBIS 2015). The UK Seabed Resources Ltd ‘UK-1’ polymetallic nodule exploration claim area is highlighted (yellow box).

The Clarion-Clipperton Zone (hereafter, CCZ) is so called as it lies between the Clarion and Clipperton Fracture Zones, topographical highs that extend longitudinally across almost the entire eastern Pacific. There is no strict definition of the region, but it has come to be regarded as the area between these fracture zones that lies within international waters and encompasses the main areas of commercial interest for polymetallic-nodule mining. Areas licensed for mining by the International Seabed Authority (ISA), as well as mining reserve areas and areas protected from mining by the ISA (ISA 2014a, Wedding et al. 2013) extend from 115°W (the easternmost extent of the UK-1 claim) to approximately 158°W, and from 22°N to 2.5°S (Fig. 1). This is an area of 6 million sq km, approximately 1.7% of the ocean’s surface.

Within the 6 million sq km CCZ, as defined above, current online data sources prior to this publication list only 50 known species of echinoderms from 290 records (OBIS 2015). This is obviously the result of lack of sampling and/or taxonomy given that an abundant and diverse echinoderm fauna is suspected in the region from photographic and video survey (e.g. Foell and Pawson 1986). The goal of the DNA taxonomy part of the ABYSSLINE program is to start to rectify these gaps in our knowledge and make data publically available that will eventually allow for a complete taxonomic synthesis of the CCZ supported by openly-available molecular and morphological data. Here we provide version 1.0 of the Echinodermata taxonomic synthesis from the ABYSSLINE program, consisting of taxon records, high-resolution imagery, genetic data from multiple markers and phylogenetic analysis from the first research cruise (AB01) aboard the RV *Melville* in October 2013. This open data publication is intended to be supported by equivalent similar data publications on the Annelida, Mollusca, Bryozoa, Cnidaria, Porifera and other taxa forming a suite of taxonomic syntheses of biodiversity in the region, supported by a contract between the company UK Seabed Resources Ltd and the Natural History Museum, London and Uni Research, Bergen, and the University of Hawaii at Manoa.

## Materials and methods

It is widely accepted that knowledge of baseline biodiversity and biogeography in the CCZ is severely hampered by a lack of modern DNA-supported taxonomic studies (ISA 2014b). With this in mind, four fundamental principles underpin our methodological pipeline: (1) A sampling design pipeline with consideration to the spatial scale of the required data, the differing biases in sampling gear and the requirement for at-sea taxonomic study, (2) A field pipeline with consideration to the successful collection of high-quality specimens using live-sorting in a 'cold-chain' from depths of 4000-5000m in the central tropical Pacific, (3) A laboratory pipeline with consideration to the needs to collect both DNA sequences and morphological data in a timely and cost-effective manner suited to the immediate needs of the science community and (4) A data and sample management pipeline that includes the publication of results with consideration to the accessibility of data and materials. Our complete methodology for DNA taxonomy in the CCZ, including deployment protocols for the various sampling gears, methods for live-sorting and microscope photography at sea and details of sample and data curation are provided in a separate open-access publication (Glover et al. 2016).

### Field pipeline

**Figure 2.**
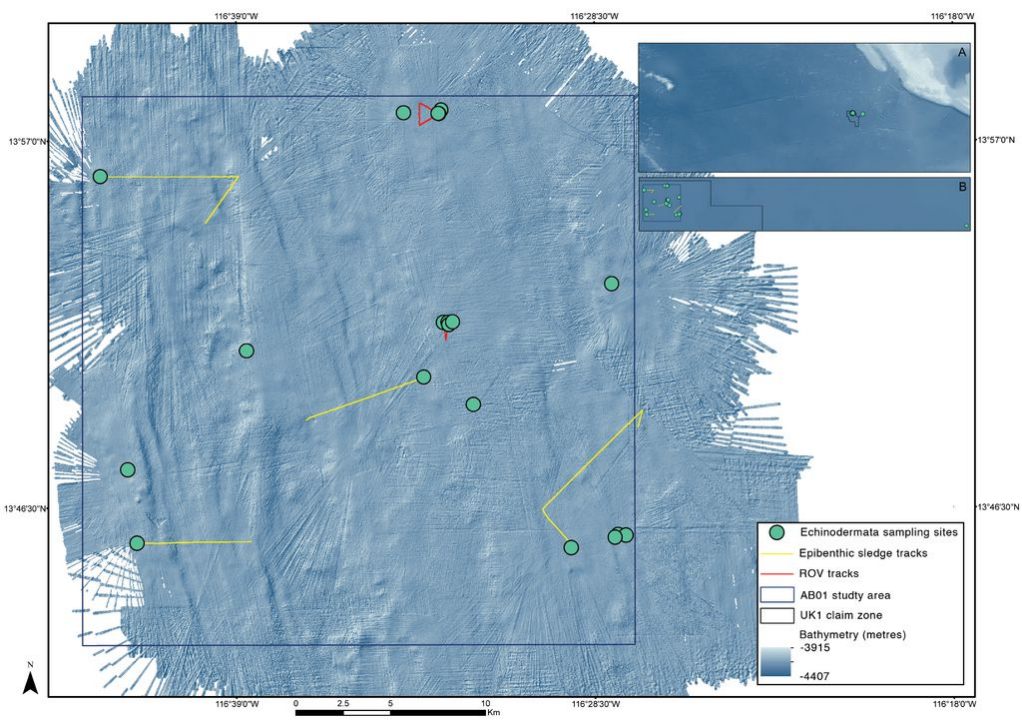
'UK-1 Stratum A' ABYSSLINE biological baseline survey box sited within the UK-1 polymetallic nodule exploration claim. Stratum A is a 30x30km survey box in the northern sector of the 58,000 km^2^ claim area. Echinoderm sample localities are indicated by green circles from the AB01 RV *Melville* survey cruise, October 2013. Inset map A: the site location within the central Pacific, inset map B: all the echinoderm sampling locations (including site 'ROV7' to the west). Both inset maps use GEBCO 2014 bathymetry (global 30 arc-second interval grid data set). Seafloor bathymetry from the RV Melville ABYSSLINE cruise is shown in the main map.

The ABYSSLINE environmental baseline survey includes three 30x30km survey boxes (strata), distributed across the UK-1 claim area, and an additional reference sites outside of the UK-1 claim area (Smith et al. 2013b, Glover et al. 2016). Within each survey stratum, sample sites for a variety of benthic sampling gears are selected randomly – a randomized, stratified sampling design that assumes no *a priori* knowledge of the benthic environment (Smith et al. 2013b). The UK-1 strata are being sampled in a series of oceanographic cruises during the course of the project, which commenced in July 2013, with the first cruise (AB01) taking place in October 2013 aboard the RV *Melville*. During this cruise, the first stratum was comprehensively mapped with multibeam bathymetry and sampled for a range of biological, environmental and geophysical parameters (Fig. 2, Smith et al. 2013).

**Figure 3.**
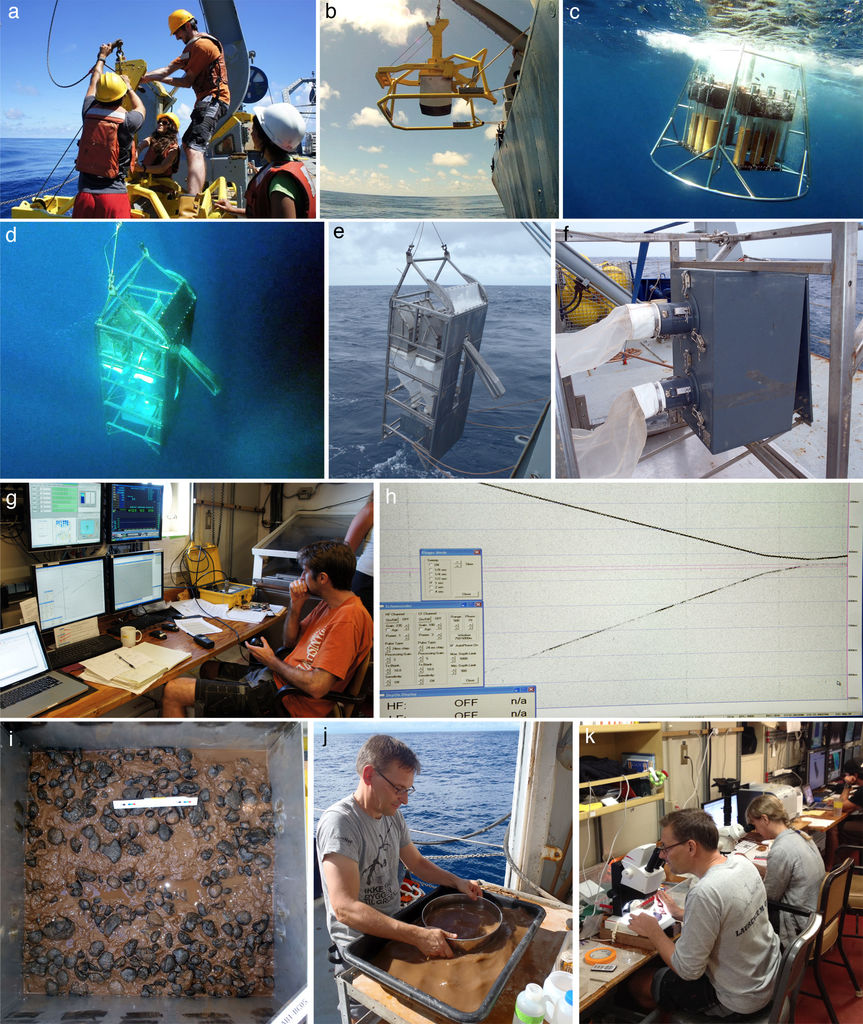
ABYSSLINE UK-1 polymetallic nodule exploration claim field pipeline for DNA taxonomy. ABYSSLINE AB01 cruise sampling aboard RV *Melville* in October 2013. (a) Preparing Box Core (BC) for deployment, (b) BC entering the water, (c) Megacore entering the water, (d-f) Epibenthic Sledge shown on recovery in water and cod-end where samples are taken, (g) controlling BC deployment on seafloor, (h) echosounder trace showing BC approaching seabed reflection, (i) successful BC surface after recovery, 50cm x 50cm, (j) carefully sifting mud in chilled filtered seawater (approx. temp 5-7°C) to remove live animals in undamaged state, (k) live-sorting aboard ship, taking samples for DNA and photomicrographs of specimens down to <1mm in size. All images by Glover, Dahlgren & Wiklund. A more comprehensive description of our methods is provided in Glover et al. 2016.

A comprehensive description of our DNA taxonomy pipeline is provided in Glover et al. 2016. In summary, deep-sea benthic specimens from the UK-1 Stratum A were collected using a range of oceanographic sampling gears including box core (BC), epibenthic sledge (EBS), remotely operated vehicle (ROV) and megacore (MC) (Fig. 2, Fig. 3). Geographic data from sampling activities were recorded on a central GIS database. Live-sorting of sediment and specimen samples was carried out aboard the RV *Melville* under the ‘cold-chain’ pipeline, in which material was immediately transferred and maintained in chilled, filtered seawater held at 2-4°C (Fig. 3). Specimens were preliminarily identified at sea and imaged live using stereomicroscopes with attached digital cameras and strobe lighting. The specimens were then transferred to individual microtube vials containing an aqueous solution of 80% non-denatured ethanol, numbered and barcoded into a database and kept chilled until return to the Natural History Museum (NHM), London. Larger, megafaunal-sized, animals were sub-sampled for DNA (with the tissue and DNA sample being taken to NHM, London) with the remaining intact specimen preserved in 10% formalin solution and taken to the University of Hawaii, Honolulu, USA for further study.

### Laboratory pipeline

Extraction of DNA was done with DNeasy Blood and Tissue Kit (Qiagen) using a Hamilton Microlab STAR Robotic Workstation. About 1800 bp of 18S, 450 bp of 16S, and 650 bp of cytochrome c oxidase subunit I (COI) were amplified using primers listed in Table 1. PCR mixtures contained 1 µl of each primer (10µM), 2 µl template DNA and 21 µl of Red Taq DNA Polymerase 1.1X MasterMix (VWR) in a mixture of total 25 µl. The PCR amplification profile consisted of initial denaturation at 95°C for 5 min, 35 cycles of denaturation at 94°C for 45 s, annealing at 55°C for 45 s, extension at 72°C for 2 min, and a final extension at 72°C for 10 min. PCR products were purified using Millipore Multiscreen 96-well PCR Purification System, and sequencing was performed on an ABI 3730XL DNA Analyser (Applied Biosystems) at The Natural History Museum Sequencing Facility, using the same primers as in the PCR reactions plus two internal primers for 18S (Table 1).

Overlapping sequence fragments were merged into consensus sequences using Geneious R6 (www.geneious.com, Kearse et al. 2012) and aligned using MAFFT (Katoh et al. 2002) for 18S and 16S, and MUSCLE (Edgar 2004) for COI, both programs used as plugins in Geneious, with default settings. Bayesian phylogenetic analyses (BA) were conducted with MrBayes 3.1.2 (Ronquist and Huelsenbeck 2003). Analyses were run for 10-20 million generations, of which 2,5-5 million generations were discarded as burn-in.

### Data pipeline

**Figure 4.**
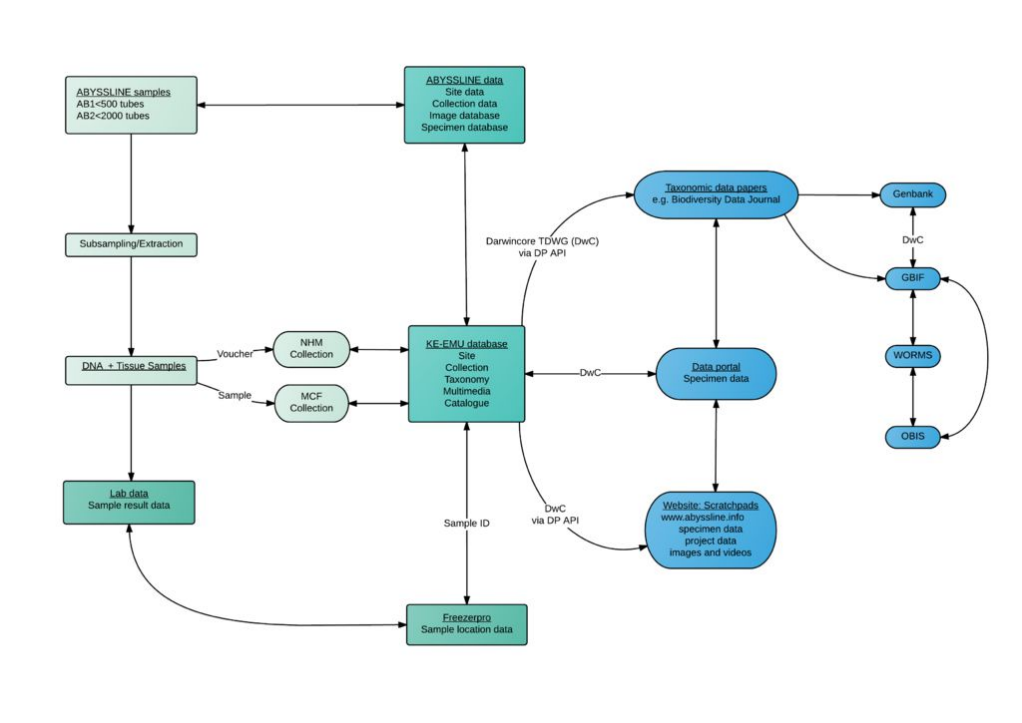
Data and sample management workflow on the ABYSSLINE DNA taxonomy project. Processes relating to a) physical samples are shown in grey, b) institution level data in dark green and c) externally-available data in blue.

The field and laboratory pipelines created a series of databases and sample sets that were then integrated into a data-management pipeline (Fig. 4). This includes the transfer and management of data and samples between a central collections database, a molecular collections database, an online scratchpad (website for faunal data) and external repositories (e.g GenBank, WoRMS, OBIS, GBIF) through a DarwinCore archive. This provides a robust data framework to support DNA taxonomy, in which openly-available data and voucher material is key to quality data standards. A further elaboration of the data pipeline is published in Glover et al. 2016

### Taxonomic assignments

All future studies of biogeographic and bathymetric ranges, gene-flow, extinction risks, natural history, reproductive ecology, functional ecology and geochemical interactions of CCZ species are dependent on accurate identifications faciliated by taxonomy. This taxonomy, presented here, is itself dependent on a sound theoretical underpinning – a species concept - coupled with the availability of both raw data and voucher samples. Here we use a phylogenetic species concept, *sensu* Donoghue 1985 with species determined by DNA-based phylogenetic analysis and the recognition of distinct monophyletic groups as species. For those taxa where the typical morphological data that allows determination of species are missing, we provide the lowest-level taxonomic name possible, but determination to species with genetic data. For species similar to a morphologically well defined species name where we lack comparable genetic data from type material or from the type locality, or when genetic data previously published in Genbank is incompatible with ours, we use the open nomenclature expression ”*cf.*". Material (including archived frozen tissue) and genetic data are accessible through the Natural History Museum, London, together with the morphological data presented in this paper, original specimens for some larger (megafaunal) taxa remain in the collection of Craig R. Smith, University of Hawaii - these specimens are indicated in the taxon treatments below. As such our species hypotheses are easily open to further evaluation and iterative improvement, e.g. full descriptions for new taxa with improved data from future cruises. A localised identification field guide to the CCZ fauna will be the subject of a future publication as more species are described, but for the present we recommend DNA-based identification (barcoding) of our species coupled with morphological comparisons made possible through this publication.

## Data resources

The following sections detail the phylogenetic analysis and data resources that underpins the species hypotheses presented in the taxon treatments. A full list of all taxa including Natural History Museum Accession Numbers, NHM Molecular Collection Facility (NHM-MCF) FreezerPro numbers and NCBI GenBank Accession numbers is provided in Table 2.

### Phylogenetic analysis of the Asteroidea

**Figure 5.**
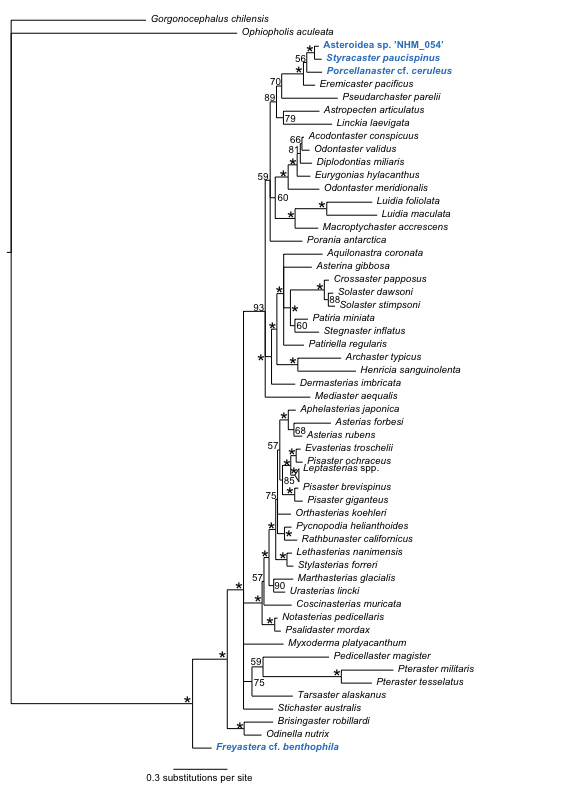
Phylogenetic analysis of the Asteroidea. 50% majority rule consensus tree from the Bayesian analyses, combining the three genes 18S, 16S and COI and using in total 60 taxa. Some of the clades are collapsed in order to make the tree smaller and easier to read.

Phylogenetic analysis of the Asteroidea (Fig. 5) reveals the presence of 4 distinct lineages of ABYSSLINE specimens which we interpret as the 4 species described below based on their genetic data.

### Phylogenetic analysis of the Crinoidea

**Figure 6.**
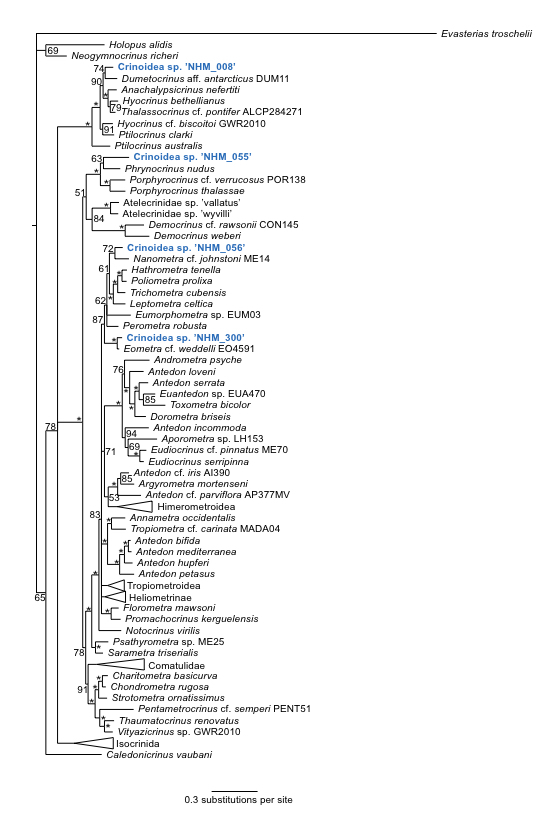
Phylogenetic analysis of the Crinoidea. 50% majority rule consensus tree from the Bayesian analyses, combining the three genes 18S, 16S and COI and using in total 113 taxa. Some of the clades are collapsed in order to make the tree smaller and easier to read.

Phylogenetic analysis of the Crinoidea (Fig. 6) reveals the presence of 4 distinct lineages of ABYSSLINE specimens which we interpret as the 4 species described below based on their genetic data.

### Phylogenetic analysis of the Holothuroidea

**Figure 7.**
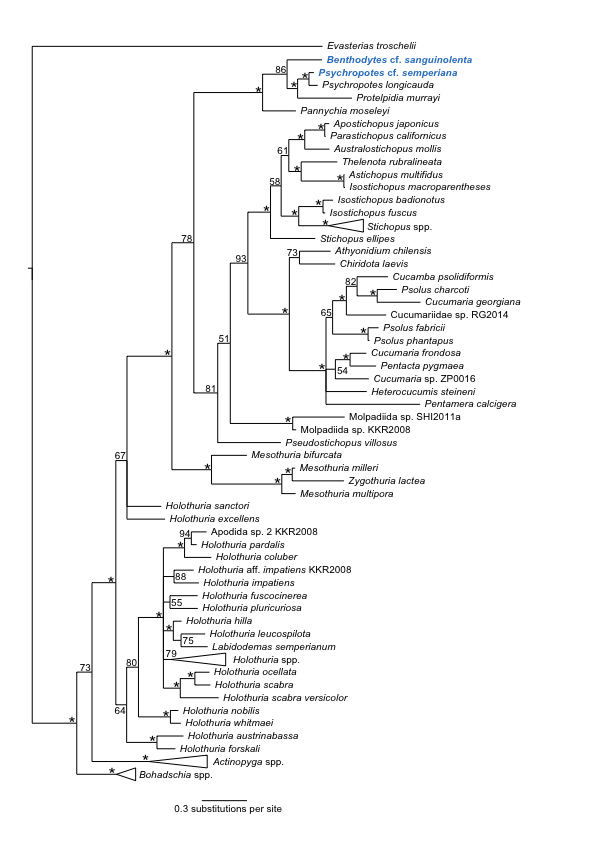
Phylogenetic analysis of the Holothuroidea. 50% majority rule consensus tree from the Bayesian analyses, using 16S and in total 115 taxa. Some of the clades are collapsed in order to make the tree smaller and easier to read.

Phylogenetic analysis of the Holothuroidea (Fig. 7) reveals the presence of 2 distinct lineages of ABYSSLINE specimens which we interpret as the 2 species described below based on their genetic data.

### Phylogenetic analysis of the Ophiuroidea

**Figure 8.**
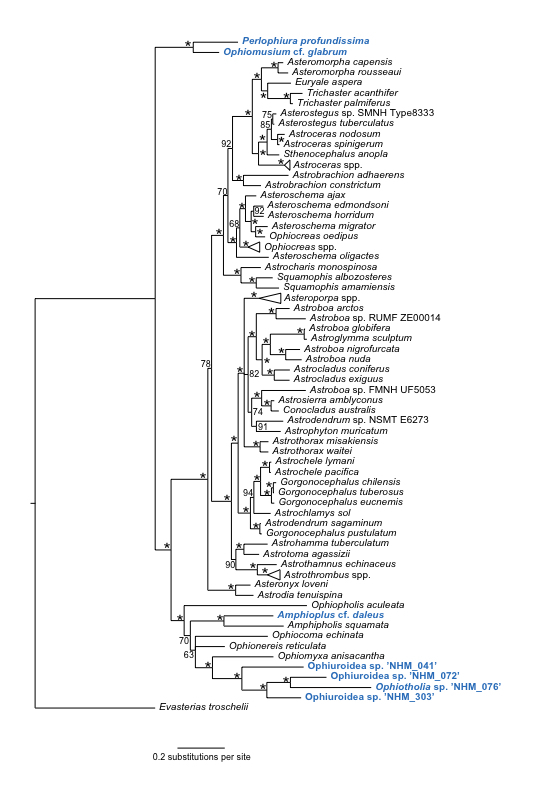
Phylogenetic analysis of the Ophiuroidea. 50% majority rule consensus tree from the Bayesian analyses, combining the three genes 18S, 16S and COI and using in total 79 taxa. Some of the clades are collapsed in order to make the tree smaller and easier to read.

Phylogenetic analysis of the Ophiuroidea (Fig. 8) the presence of 7 distinct lineages of ABYSSLINE specimens which we interpret as the 7 species described below based on their genetic data.

## Taxon treatments

### Asteroidea sp. 'NHM_054'

#### Materials

- **Type status:** Other material. **Occurrence:** catalogNumber: de4bd6ce-07fe-496e-bffc-67a4c6b9782c; recordNumber: NHM_054; recordedBy: Adrian Glover, Helena Wiklund, Thomas Dahlgren, Maggie Georgieva; individualCount: 1; preparations: tissue and DNA voucher stored in 80% non-denatured ethanol aqueous solution; otherCatalogNumbers: 5023476; associatedSequences: http://www.ncbi.nlm.nih.gov/nuccore/KU519512 | KU519530; **Taxon:** taxonConceptID: Asteroidea sp. (NHM_054); scientificName: Asteroidea; kingdom: Animalia; phylum: Echinodermata; class: Asteroidea; scientificNameAuthorship: de Blainville, 1830; **Location:** waterBody: Pacific; stateProvince: Clarion Clipperton Zone; locality: UK Seabed Resources Ltd exploration claim UK-1; verbatimLocality: UK-1 Stratum A; maximumDepthInMeters: 4108; locationRemarks: RV Melville Cruise MV1313; decimalLatitude: 13.849883333333; decimalLongitude: -116.64495; geodeticDatum: WGS84; **Identification:** identifiedBy: Adrian Glover, Helena Wiklund, Thomas Dahlgren; dateIdentified: 2015-06-01; identificationRemarks: identified by DNA and morphology; **Event:** samplingProtocol: USNEL Box Core; eventDate: 2013-10-09; eventTime: 17:34; habitat: Abyssal plain; fieldNumber: BC04; fieldNotes: Collected from 0-2 cm layer of box core using a 300 micron sieve; **Record Level:** language: en; institutionCode: NHMUK; collectionCode: ZOO; datasetName: ABYSSLINE; basisOfRecord: PreservedSpecimen
- **Type status:** Other material. **Occurrence:** catalogNumber: bc03fc1a-3613-41a2-b1f1-bf905e0fa6d0; recordNumber: NHM_375; recordedBy: Adrian Glover, Helena Wiklund, Thomas Dahlgren, Maggie Georgieva; individualCount: 1; preparations: tissue and DNA voucher stored in 80% non-denatured ethanol aqueous solution; otherCatalogNumbers: 5023517; associatedSequences: http://www.ncbi.nlm.nih.gov/nuccore/KU519528; **Taxon:** taxonConceptID: Asteroidea sp. (NHM_054); scientificName: Asteroidea; kingdom: Animalia; phylum: Echinodermata; class: Asteroidea; scientificNameAuthorship: de Blainville, 1830; **Location:** waterBody: Pacific; stateProvince: Clarion Clipperton Zone; locality: UK Seabed Resources Ltd exploration claim UK-1; verbatimLocality: UK-1 Stratum A; maximumDepthInMeters: 4182; locationRemarks: RV Melville Cruise MV1313; decimalLatitude: 13.933066666667; decimalLongitude: -116.71628333333; geodeticDatum: WGS84; **Identification:** identifiedBy: Adrian Glover, Helena Wiklund, Thomas Dahlgren; dateIdentified: 2015-06-01; identificationRemarks: identified by DNA and morphology; **Event:** samplingProtocol: Brenke Epibenthic Sledge; eventDate: 2013-10-19; eventTime: 12:16; habitat: Abyssal plain; fieldNumber: EB05; fieldNotes: Collected from epi net (on the epibenthic sledge); **Record Level:** language: en; institutionCode: NHMUK; collectionCode: ZOO; datasetName: ABYSSLINE; basisOfRecord: PreservedSpecimen

#### Description

**Figure 9.**
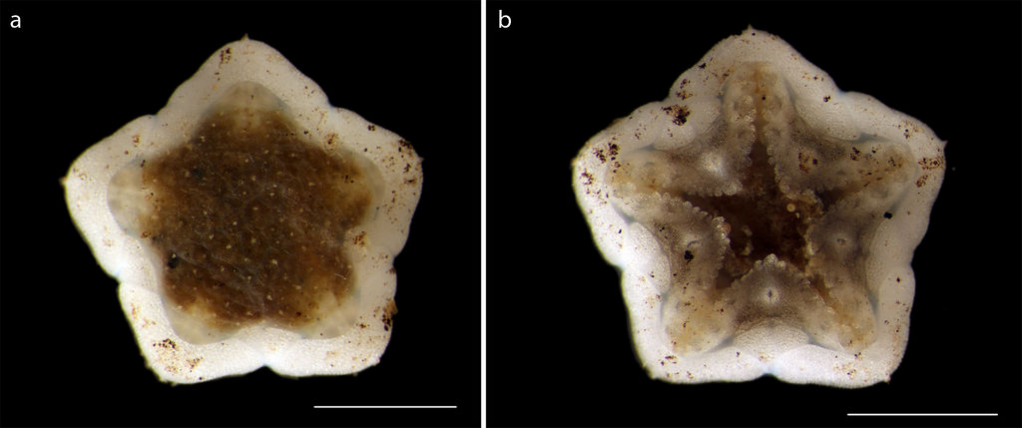
Asteroidea sp. Specimen NHM_054. (a) Dorsal. (b) Ventral. Scale bars (a,b) 2mm. Image attribution Glover, Dahlgren & Wiklund 2015.

Voucher material NHM_54, width of disc 4.7mm. Arms absent. Medial antenna absent (Fig. 9).

Genetic data for this taxa with new GenBank accession numbers are provided in Table 2

#### Diagnosis

Morphologically and genetically close to *Eremicaster* sp (Fig. 5). Forms a unique monophyletic clade distinct from other AB01 specimens. No genetic matches on GenBank or Barcode of Life Database.

### Freyastera benthophila

(Sladen, 1889)

#### Materials

- **Type status:** Other material. **Occurrence:** catalogNumber: b7ffe7a2-7be1-4d4f-b784-7aaecf0ee743; recordNumber: NHM_413; recordedBy: Adrian Glover, Helena Wiklund, Thomas Dahlgren, Maggie Georgieva; individualCount: 1; preparations: tissue and DNA voucher stored in 80% non-denatured ethanol aqueous solution; otherCatalogNumbers: 5023520; associatedSequences: http://www.ncbi.nlm.nih.gov/nuccore/KU519550 | KU519518 | KU519535; **Taxon:** taxonConceptID: Freyastera cf. benthophila; scientificName: Freyastera benthophila; kingdom: Animalia; phylum: Echinodermata; class: Asteroidea; order: Brisingida; family: Freyellidae; genus: Freyastera; scientificNameAuthorship: (Sladen, 1889); **Location:** waterBody: Pacific; stateProvince: Clarion Clipperton Zone; locality: UK Seabed Resources Ltd exploration claim UK-1; verbatimLocality: UK-1 Stratum A; maximumDepthInMeters: 4011; locationRemarks: RV Melville Cruise MV1313; decimalLatitude: 13.862225; decimalLongitude: -116.546215; geodeticDatum: WGS84; **Identification:** identifiedBy: Diva Amon, Chris Mah, Adrian Glover, Helena Wiklund, Thomas Dahlgren; dateIdentified: 2015-06-01; identificationRemarks: identified by DNA and morphology; identificationQualifier: cf; **Event:** samplingProtocol: Remotely Operated Vehicle; eventDate: 2013-10-21; eventTime: 00:39; habitat: Abyssal plain; fieldNumber: RV06; **Record Level:** language: en; institutionCode: NHMUK; collectionCode: ZOO; datasetName: ABYSSLINE; basisOfRecord: PreservedSpecimen
- **Type status:** Other material. **Occurrence:** catalogNumber: 16599946-2aba-4710-98e6-43c522061878; recordNumber: NHM_421; recordedBy: Adrian Glover, Helena Wiklund, Thomas Dahlgren, Maggie Georgieva; individualCount: 3; preparations: tissue and DNA voucher stored in 80% non-denatured ethanol aqueous solution; otherCatalogNumbers: 5023523; associatedSequences: http://www.ncbi.nlm.nih.gov/nuccore/KU519551; **Taxon:** taxonConceptID: Freyastera cf. benthophila; scientificName: Freyastera benthophila; kingdom: Animalia; phylum: Echinodermata; class: Asteroidea; order: Brisingida; family: Freyellidae; genus: Freyastera; scientificNameAuthorship: (Sladen, 1889); **Location:** waterBody: Pacific; stateProvince: Clarion Clipperton Zone; locality: UK Seabed Resources Ltd exploration claim UK-1; verbatimLocality: UK-1 Stratum A; maximumDepthInMeters: 4011; locationRemarks: RV Melville Cruise MV1313; decimalLatitude: 13.862225; decimalLongitude: -116.546215; geodeticDatum: WGS84; **Identification:** identifiedBy: Diva Amon, Chris Mah, Adrian Glover, Helena Wiklund, Thomas Dahlgren; dateIdentified: 2015-06-01; identificationRemarks: identified by DNA and morphology; identificationQualifier: cf; **Event:** samplingProtocol: Remotely Operated Vehicle; eventDate: 2013-10-21; eventTime: 00:39; habitat: Abyssal plain; fieldNumber: RV06; **Record Level:** language: en; institutionCode: NHMUK; collectionCode: ZOO; datasetName: ABYSSLINE; basisOfRecord: PreservedSpecimen

#### Description

**Figure 10.**
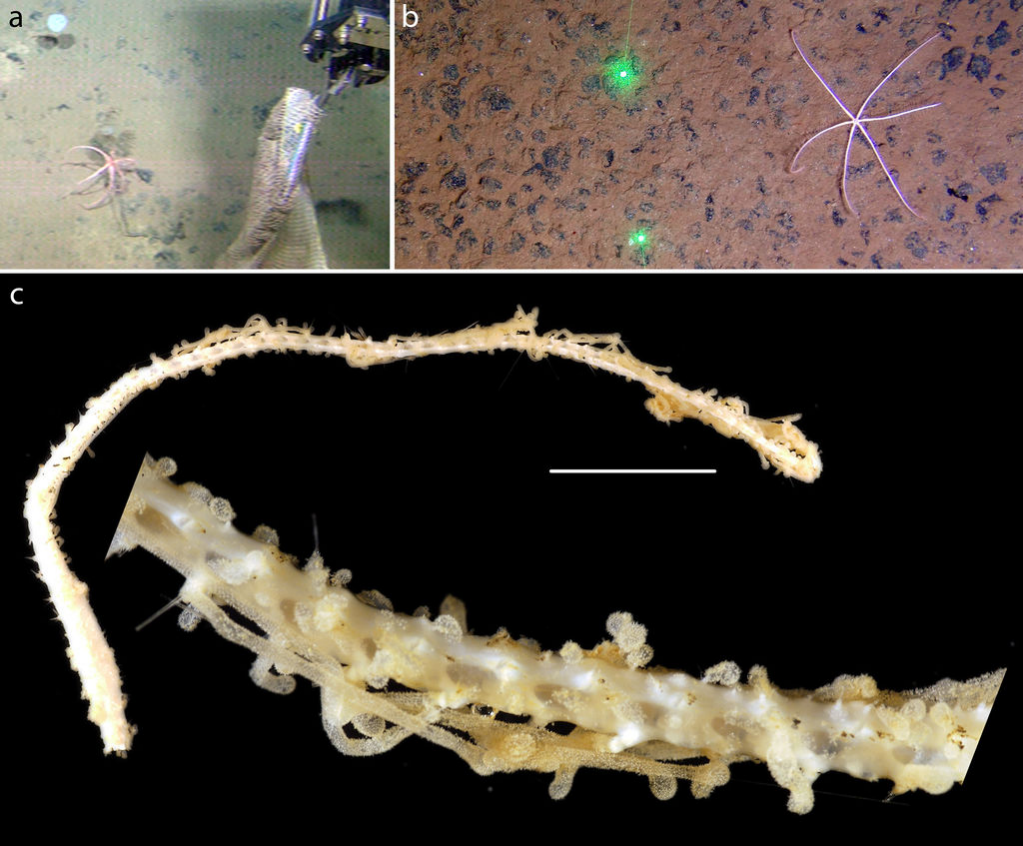
Freyastera cf. benthophila Sladen, 1899. (a) Specimen NHM_413 (arm fragment) being recovered *in situ* from the seafloor during ROV dive RV06, (b) Additional, unsampled specimen, imaged during AB01 video survey and identified based on imagery as the same species, (c) Tentacle of specimen NHM_413 only part recovered; inset shows detail of tentacle. Scale bars (b) laser dots 242mm apart, (c) 20mm. Image attribution (a) Smith & Amon 2013, (c) Glover, Dahlgren & Wiklund 2015.

 15cm long arm fragments of a Freyellidae recovered from ROV biobox. Identified by DNA and morphological examination (Fig. 10). Morphological identification suggests *Freyastera benthophila* detailed in Sladen 1889​).

Genetic data for this taxa with new GenBank accession numbers are provided in Table 2

#### Diagnosis

Forms a unique monophyletic clade distinct from other AB01 specimens (Fig. 5). The speciemens differs significantly in sequence identity to the published 16S sequence of *Freyastera benthophila* on GenBank accession EU722993 (K2P = 0,064). The type locality of *Freyastera benthophila* is in South Pacific (39°41'S; 131°23'W, 4663m depth).

### Porcellanaster ceruleus

Wyville Thomson, 1877

#### Materials

- **Type status:** Other material. **Occurrence:** catalogNumber: c57f1bd3-1b32-41e6-8e1d-0ad6472e4327; recordNumber: NHM_168; recordedBy: Adrian Glover, Helena Wiklund, Thomas Dahlgren, Maggie Georgieva; individualCount: 1; preparations: tissue and DNA voucher stored in 80% non-denatured ethanol aqueous solution; otherCatalogNumbers: 5023491; associatedSequences: http://www.ncbi.nlm.nih.gov/nuccore/KU519568; **Taxon:** taxonConceptID: Porcellanaster cf. ceruleus; scientificName: Porcellanaster ceruleus; kingdom: Animalia; phylum: Echinodermata; class: Asteroidea; order: Paxillosida; family: Porcellanasteridae; genus: Porcellanaster ; scientificNameAuthorship: Wyville Thomson, 1877; **Location:** waterBody: Pacific; stateProvince: Clarion Clipperton Zone; locality: UK Seabed Resources Ltd exploration claim UK-1; verbatimLocality: UK-1 Stratum A; maximumDepthInMeters: 4084; locationRemarks: RV Melville Cruise MV1313; decimalLatitude: 13.963233333333; decimalLongitude: -116.56821666667; geodeticDatum: WGS84; **Identification:** identifiedBy: Diva Amon, Chris Mah, Adrian Glover, Helena Wiklund, Thomas Dahlgren; dateIdentified: 2015-06-01; identificationRemarks: identified by DNA and morphology; identificationQualifier: cf; **Event:** samplingProtocol: USNEL Box Core; eventDate: 2013-10-12; eventTime: 23:01; habitat: Abyssal plain; fieldNumber: BC06; fieldNotes: Collected from 0-2 cm layer of box core using a 300 micron sieve; **Record Level:** language: en; institutionCode: NHMUK; collectionCode: ZOO; datasetName: ABYSSLINE; basisOfRecord: PreservedSpecimen
- **Type status:** Other material. **Occurrence:** catalogNumber: 7e8ca2d8-aea1-45bd-b7e0-d0575cadd82d; recordNumber: NHM_200; recordedBy: Adrian Glover, Helena Wiklund, Thomas Dahlgren, Maggie Georgieva; individualCount: 1; preparations: tissue and DNA voucher stored in 80% non-denatured ethanol aqueous solution; otherCatalogNumbers: 5023495; associatedSequences: http://www.ncbi.nlm.nih.gov/nuccore/KU519569; **Taxon:** taxonConceptID: Porcellanaster cf. ceruleus; scientificName: Porcellanaster ceruleus; kingdom: Animalia; phylum: Echinodermata; class: Asteroidea; order: Paxillosida; family: Porcellanasteridae; genus: Porcellanaster ; scientificNameAuthorship: Wyville Thomson, 1877; **Location:** waterBody: Pacific; stateProvince: Clarion Clipperton Zone; locality: UK Seabed Resources Ltd exploration claim UK-1; verbatimLocality: UK-1 Stratum A; maximumDepthInMeters: 4054; locationRemarks: RV Melville Cruise MV1313; decimalLatitude: 13.824116666667; decimalLongitude: -116.53425; geodeticDatum: WGS84; **Identification:** identifiedBy: Diva Amon, Chris Mah, Adrian Glover, Helena Wiklund, Thomas Dahlgren; dateIdentified: 2015-06-01; identificationRemarks: identified by DNA and morphology; identificationQualifier: cf; **Event:** samplingProtocol: USNEL Box Core; eventDate: 2013-10-14; eventTime: 21:37; habitat: Abyssal plain; fieldNumber: BC07; fieldNotes: Collected from 2-5 cm layer of box core using a 300 micron sieve; **Record Level:** language: en; institutionCode: NHMUK; collectionCode: ZOO; datasetName: ABYSSLINE; basisOfRecord: PreservedSpecimen
- **Type status:** Other material. **Occurrence:** catalogNumber: 95d0bd7f-0df9-47e4-8003-cd12007d54b4; recordNumber: NHM_253; recordedBy: Adrian Glover, Helena Wiklund, Thomas Dahlgren, Maggie Georgieva; individualCount: 2; preparations: tissue and DNA voucher stored in 80% non-denatured ethanol aqueous solution; otherCatalogNumbers: 5023506; associatedSequences: http://www.ncbi.nlm.nih.gov/nuccore/KU519570 | KU519525 | KU519542; **Taxon:** taxonConceptID: Porcellanaster cf. ceruleus; scientificName: Porcellanaster ceruleus; kingdom: Animalia; phylum: Echinodermata; class: Asteroidea; order: Paxillosida; family: Porcellanasteridae; genus: Porcellanaster ; scientificNameAuthorship: Wyville Thomson, 1877; **Location:** waterBody: Pacific; stateProvince: Clarion Clipperton Zone; locality: UK Seabed Resources Ltd exploration claim UK-1; verbatimLocality: UK-1 Stratum A; maximumDepthInMeters: 4076; locationRemarks: RV Melville Cruise MV1313; decimalLatitude: 13.755833333333; decimalLongitude: -116.48666666667; geodeticDatum: WGS84; **Identification:** identifiedBy: Diva Amon, Chris Mah, Adrian Glover, Helena Wiklund, Thomas Dahlgren; dateIdentified: 2015-06-01; identificationRemarks: identified by DNA and morphology; identificationQualifier: cf; **Event:** samplingProtocol: Brenke Epibenthic Sledge; eventDate: 2013-10-17; eventTime: 01:50; habitat: Abyssal plain; fieldNumber: EB04; fieldNotes: Collected from epi net (on the epibenthic sledge); **Record Level:** language: en; institutionCode: NHMUK; collectionCode: ZOO; datasetName: ABYSSLINE; basisOfRecord: PreservedSpecimen
- **Type status:** Other material. **Occurrence:** catalogNumber: d15a68e0-b2b3-40b4-8cab-0563609cc80d; recordNumber: NHM_267; recordedBy: Adrian Glover, Helena Wiklund, Thomas Dahlgren, Maggie Georgieva; individualCount: 1; preparations: tissue and DNA voucher stored in 80% non-denatured ethanol aqueous solution; otherCatalogNumbers: 5023509; associatedSequences: http://www.ncbi.nlm.nih.gov/nuccore/KU519571; **Taxon:** taxonConceptID: Porcellanaster cf. ceruleus; scientificName: Porcellanaster ceruleus; kingdom: Animalia; phylum: Echinodermata; class: Asteroidea; order: Paxillosida; family: Porcellanasteridae; genus: Porcellanaster ; scientificNameAuthorship: Wyville Thomson, 1877; **Location:** waterBody: Pacific; stateProvince: Clarion Clipperton Zone; locality: UK Seabed Resources Ltd exploration claim UK-1; verbatimLocality: UK-1 Stratum A; maximumDepthInMeters: 4076; locationRemarks: RV Melville Cruise MV1313; decimalLatitude: 13.755833333333; decimalLongitude: -116.48666666667; geodeticDatum: WGS84; **Identification:** identifiedBy: Diva Amon, Chris Mah, Adrian Glover, Helena Wiklund, Thomas Dahlgren; dateIdentified: 2015-06-01; identificationRemarks: identified by DNA and morphology; identificationQualifier: cf; **Event:** samplingProtocol: Brenke Epibenthic Sledge; eventDate: 2013-10-17; eventTime: 01:50; habitat: Abyssal plain; fieldNumber: EB04; fieldNotes: Collected from epi net (on the epibenthic sledge); **Record Level:** language: en; institutionCode: NHMUK; collectionCode: ZOO; datasetName: ABYSSLINE; basisOfRecord: PreservedSpecimen
- **Type status:** Other material. **Occurrence:** catalogNumber: 76acc5a2-6e0e-4599-8104-b8e243af10c4; recordNumber: NHM_408; recordedBy: Adrian Glover, Helena Wiklund, Thomas Dahlgren, Maggie Georgieva; individualCount: 1; preparations: tissue and DNA voucher stored in 80% non-denatured ethanol aqueous solution; otherCatalogNumbers: 5023519; associatedSequences: http://www.ncbi.nlm.nih.gov/nuccore/KU519572; **Taxon:** taxonConceptID: Porcellanaster cf. ceruleus; scientificName: Porcellanaster ceruleus; kingdom: Animalia; phylum: Echinodermata; class: Asteroidea; order: Paxillosida; family: Porcellanasteridae; genus: Porcellanaster ; scientificNameAuthorship: Wyville Thomson, 1877; **Location:** waterBody: Pacific; stateProvince: Clarion Clipperton Zone; locality: UK Seabed Resources Ltd exploration claim UK-1; verbatimLocality: UK-1 Stratum A; maximumDepthInMeters: 4500; locationRemarks: RV Melville Cruise MV1313; decimalLatitude: 13.863283333333; decimalLongitude: -116.54885; geodeticDatum: WGS84; **Identification:** identifiedBy: Diva Amon, Chris Mah, Adrian Glover, Helena Wiklund, Thomas Dahlgren; dateIdentified: 2015-06-01; identificationRemarks: identified by DNA and morphology; identificationQualifier: cf; **Event:** samplingProtocol: USNEL Box Core; eventDate: 2013-10-20; eventTime: 03:39; habitat: Abyssal plain; fieldNumber: BC12; fieldNotes: Collected from 0-2 cm layer of box core using a 300 micron sieve; **Record Level:** language: en; institutionCode: NHMUK; collectionCode: ZOO; datasetName: ABYSSLINE; basisOfRecord: PreservedSpecimen

#### Description

**Figure 11.**
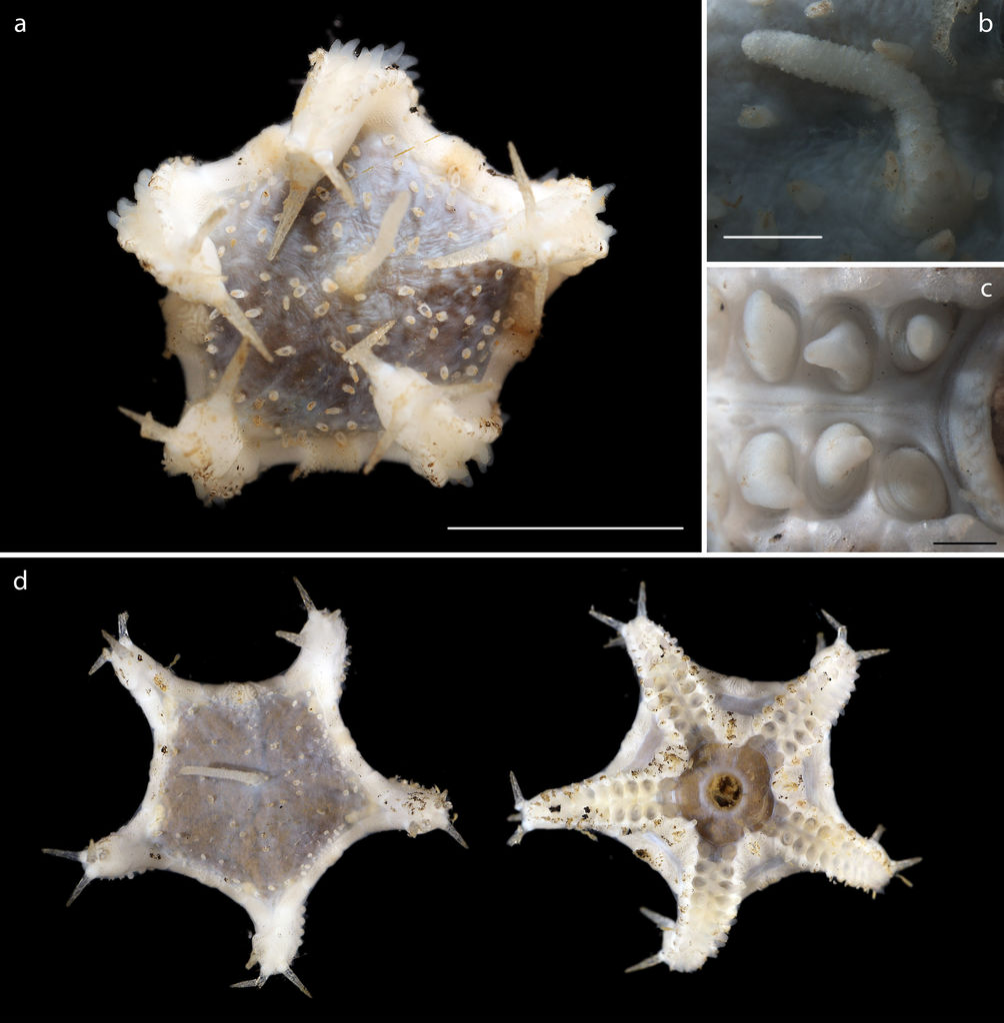
Porcellanaster cf. ceruleus Wyville Thomson, 1877 (a) Specimen NHM_267. (b) Detail of medial antenna. (c) Detail of tube feet. (d) Specimen NHM_253. Scale bars (a) 5mm, (b) 1mm, (c) 0.5mm. Image attribution Glover, Dahlgren & Wiklund 2015.

Voucher material NHM_267 maximum width of disc 10.5mm (Fig. 11). Length of medial antenna 3.1mm- specimen NHM_253 (Fig. 11). Morphological identification matches *Porcellanaster ceruleus* detailed in Wywille Thomson (1877).

Genetic data for this taxa with new GenBank accession numbers are provided in Table 2

#### Diagnosis

Morphologically matches diagnosis of *Porcellanaster ceruleus* Wyville Thomson, 1877. Forms a unique monophyletic clade distinct from other AB01 specimens. Sequences of this material has no genetic matches on GenBank or Barcode of Life Database. The type material of *Porcellanaster ceruleus* Wyville Thomson, 1877 was dredged by the Challanger SE of New York (38°34'N; 72°10'W, 2270m depth) wich is significantly separated from our collection site. We assign the tentative name Porcellanaster cf. ceruleus to this material until we have a better understanding of genetic variation within the species including data from the type locality.

### Styracaster paucispinus

Ludwig, 1907

#### Materials

- **Type status:** Other material. **Occurrence:** catalogNumber: 4ae2430e-549e-47f2-ba5d-0e9a08443d31; recordNumber: NHM_374; recordedBy: Adrian Glover, Helena Wiklund, Thomas Dahlgren, Maggie Georgieva; individualCount: 1; preparations: tissue and DNA voucher stored in 80% non-denatured ethanol aqueous solution; otherCatalogNumbers: 5023516; associatedSequences: http://www.ncbi.nlm.nih.gov/nuccore/KU519573 | KU519527 | KU519543; **Taxon:** taxonConceptID: Styracaster paucispinus; scientificName: Styracaster paucispinus; kingdom: Animalia; phylum: Echinodermata; class: Asteroidea; order: Paxillosida; family: Porcellanasteridae; genus: Styracaster; scientificNameAuthorship: Ludwig, 1907; **Location:** waterBody: Pacific; stateProvince: Clarion Clipperton Zone; locality: UK Seabed Resources Ltd exploration claim UK-1; verbatimLocality: UK-1 Stratum A; maximumDepthInMeters: 4182; locationRemarks: RV Melville Cruise MV1313; decimalLatitude: 13.933066666667; decimalLongitude: -116.71628333333; geodeticDatum: WGS84; **Identification:** identifiedBy: Diva Amon, Chris Mah, Adrian Glover, Helena Wiklund, Thomas Dahlgren; dateIdentified: 2015-06-01; identificationRemarks: identified by DNA and morphology; **Event:** samplingProtocol: Brenke Epibenthic Sledge; eventDate: 2013-10-19; eventTime: 12:16; habitat: Abyssal plain; fieldNumber: EB05; fieldNotes: Collected from epi net (on the epibenthic sledge); **Record Level:** language: en; institutionCode: NHMUK; collectionCode: ZOO; datasetName: ABYSSLINE; basisOfRecord: PreservedSpecimen

#### Description

**Figure 12.**
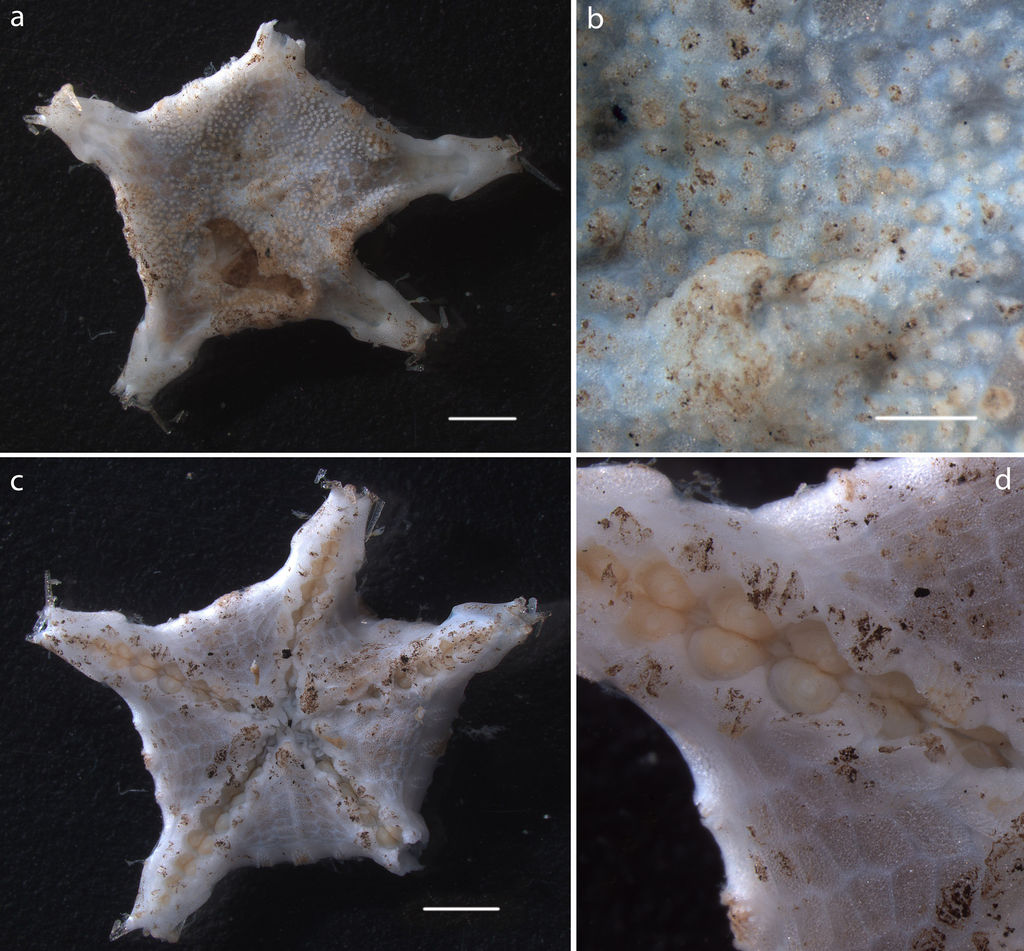
*Styracaster paucispinus* Ludwig, 1907. Specimen NHM_374 (a) Dorsal. (b) Detail of dorsal surface. (c) Ventral. (d) Ventral feet. Scale bars (a) 2mm, (b) 0.5mm, (c) 2mm. Image attribution Glover, Dahlgren & Wiklund 2015

Voucher material NHM_374, width of disc 8.2mm, maximum width of specimen including arms 16.5mm (Fig. 12).

Genetic data for this taxa with new GenBank accession numbers are provided in Table 2

#### Diagnosis

Morphologically matches diagnosis of *Styracaster paucispinus* based on descriptions in Madsen 1961, Ludwig 1905 and material from the USNM collections with taxonomic adjustments from Mah 2015. Forms a unique monophyletic clade distinct from other AB01 specimens. No genetic matches on GenBank or Barcode of Life Database. The type material was collected in the Pacific Ocean at a similar depth to our material (8°30'S; 85°36'W, 4300m depth).

### Crinoidea sp. 'NHM_008'

#### Materials

- **Type status:** Other material. **Occurrence:** catalogNumber: b2a871bf-46d5-4639-a839-427a3efa848c; recordNumber: NHM_008; recordedBy: Adrian Glover, Helena Wiklund, Thomas Dahlgren, Maggie Georgieva; individualCount: 1; preparations: tissue and DNA voucher stored in 80% non-denatured ethanol aqueous solution; otherCatalogNumbers: 5023472; associatedSequences: http://www.ncbi.nlm.nih.gov/nuccore/KU519547 | KU519514 | KU519531; **Taxon:** taxonConceptID: Crinoidea sp. (NHM_008); scientificName: Crinoidea; kingdom: Animalia; phylum: Echinodermata; class: Crinoidea; **Location:** waterBody: Pacific; stateProvince: Clarion Clipperton Zone; locality: UK Seabed Resources Ltd exploration claim UK-1; verbatimLocality: UK-1 Stratum A; maximumDepthInMeters: 4171; locationRemarks: RV Melville Cruise MV1313; decimalLatitude: 13.881666666667; decimalLongitude: -116.46666666667; geodeticDatum: WGS84; **Identification:** identifiedBy: Adrian Glover, Helena Wiklund, Thomas Dahlgren; dateIdentified: 2015-06-01; identificationRemarks: identified by DNA and morphology; **Event:** samplingProtocol: USNEL Box Core; eventDate: 2013-10-08; eventTime: 17:15; habitat: Abyssal plain; fieldNumber: BC03; fieldNotes: Collected from polymetallic nodule found in benthic sediment; **Record Level:** language: en; institutionCode: NHMUK; collectionCode: ZOO; datasetName: ABYSSLINE; basisOfRecord: PreservedSpecimen

#### Description

**Figure 13.**
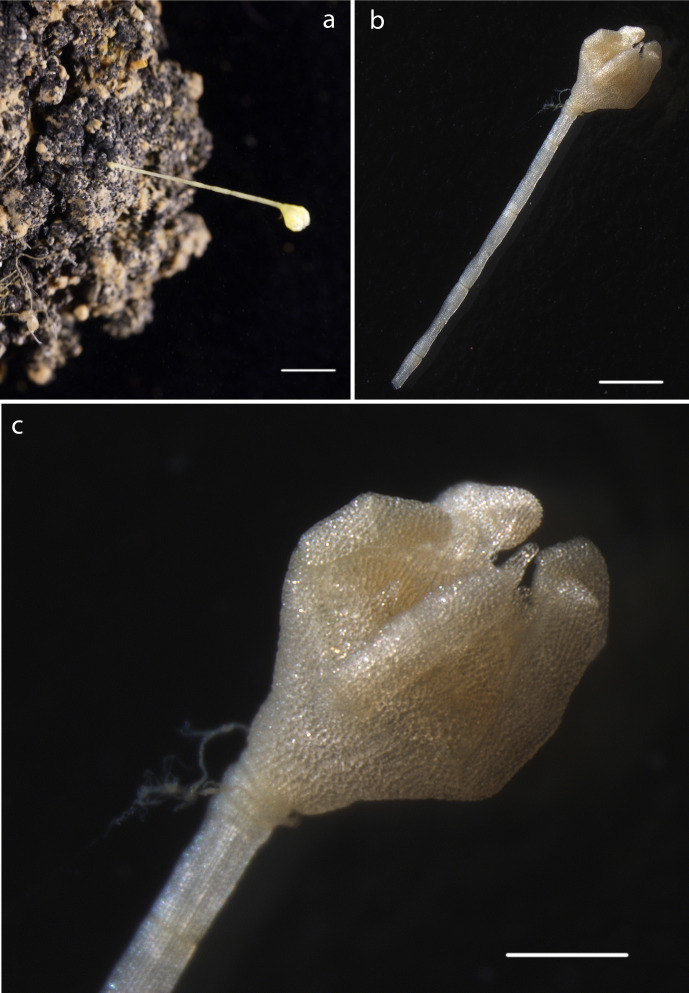
Crinoidea sp. Specimen NHM_008. (a) Specimen attached to polymetallic nodule, live photograph, after recovery from box core. (b) Preserved specimen following DNA extraction. (c) Detail of crown, calyx and basals. Scale bars (a) 3mm, (b) 1mm, (c) 0.5mm. Image attribution Glover, Dahlgren & Wiklund 2015.

Calyx 1.5mm long and 1.4mm wide with arms possibly incomplete. Arms present with 0.24mm in width, 0.95mm in length. Total length of calyx and distal part of stalk preserved 6.5mm. Stalk 0.32mm in width, stalk columnals approx 1mm in length. (Fig. 13)

Genetic data for this taxa with new GenBank accession numbers are provided in Table 2

#### Diagnosis

Morphologically close to *Hyocrinus foelli* Roux and Pawson 1999 but incomplete specimen prevents full identification. Forms a unique monophyletic clade distinct from other AB01 specimens. No genetic matches on GenBank or Barcode of Life Database.

#### Ecology

Specimen observed live on a small potato-sized polymetallic nodule from the eastern CCZ abyssal plain.

### Crinoidea sp. 'NHM_055'

#### Materials

- **Type status:** Other material. **Occurrence:** catalogNumber: 280c758b-5287-4a13-9f45-f6a6150b37d0; recordNumber: NHM_055; recordedBy: Adrian Glover, Helena Wiklund, Thomas Dahlgren, Maggie Georgieva; individualCount: 2; preparations: tissue and DNA voucher stored in 80% non-denatured ethanol aqueous solution; otherCatalogNumbers: 5023477; associatedSequences: http://www.ncbi.nlm.nih.gov/nuccore/KU519548 | KU519515 | KU519532; **Taxon:** taxonConceptID: Crinoidea sp. (NHM_055); scientificName: Crinoidea; kingdom: Animalia; phylum: Echinodermata; class: Crinoidea; **Location:** waterBody: Pacific; stateProvince: Clarion Clipperton Zone; locality: UK Seabed Resources Ltd exploration claim UK-1; verbatimLocality: UK-1 Stratum A; maximumDepthInMeters: 4108; locationRemarks: RV Melville Cruise MV1313; decimalLatitude: 13.849883333333; decimalLongitude: -116.64495; geodeticDatum: WGS84; **Identification:** identifiedBy: Adrian Glover, Helena Wiklund, Thomas Dahlgren; dateIdentified: 2015-06-01; identificationRemarks: identified by DNA and morphology; **Event:** samplingProtocol: USNEL Box Core; eventDate: 2013-10-09; eventTime: 17:34; habitat: Abyssal plain; fieldNumber: BC04; fieldNotes: Collected from polymetallic nodule found in benthic sediment; **Record Level:** language: en; institutionCode: NHMUK; collectionCode: ZOO; datasetName: ABYSSLINE; basisOfRecord: PreservedSpecimen

#### Description

**Figure 14.**
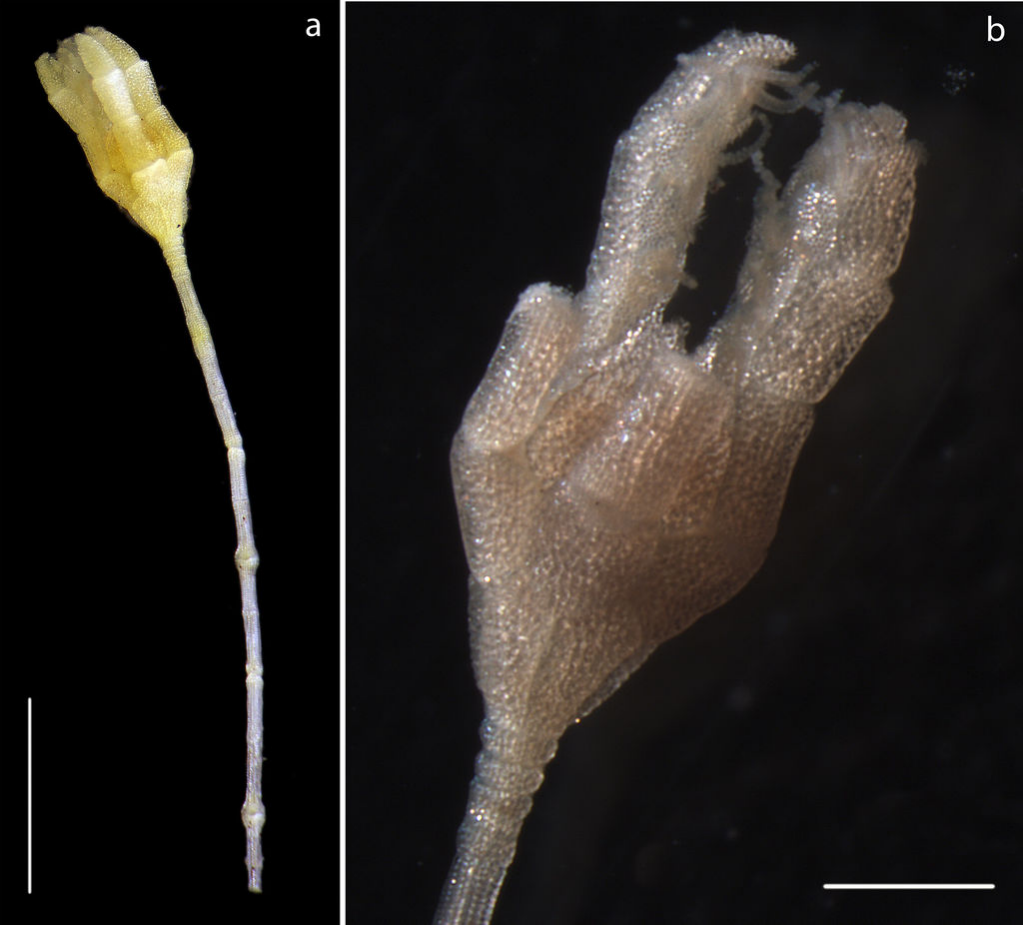
Crinoidea sp. Specimen NHM_055. (a) Specimen after removal from polymetallic nodule. (b) Detail of crown, calyx and arms as present after DNA extraction from 2 arms. Scale bars (a) 2mm, (b) 0.5mm. Image attribution Glover, Dahlgren & Wiklund 2015

Specimen including stalk and crown, calyx with arms, 8mm in total length, 5 arms, 0.31mm in width, as present in original specimen, prior to DNA sampling, with length 1.3mm from distal portion of calyx. Distally, pinnules observed on arms (Fig. 14).

Genetic data for this taxa with new GenBank accession numbers are provided in Table 2

#### Diagnosis

Forms a unique monophyletic clade distinct from other AB01 specimens. No genetic matches on GenBank or Barcode of Life Database.

#### Ecology

Found living on polymetallic nodule.

### Crinoidea sp. 'NHM_056'

#### Materials

- **Type status:** Other material. **Occurrence:** catalogNumber: 92825c07-a16d-4c5e-a8e9-4fbcdc8cf44a; recordNumber: NHM_056; recordedBy: Adrian Glover, Helena Wiklund, Thomas Dahlgren, Maggie Georgieva; individualCount: 1; preparations: tissue and DNA voucher stored in 80% non-denatured ethanol aqueous solution; otherCatalogNumbers: 5023479; associatedSequences: http://www.ncbi.nlm.nih.gov/nuccore/KU519516 | KU519533; **Taxon:** taxonConceptID: Crinoidea sp. (NHM_056); scientificName: Crinoidea; kingdom: Animalia; phylum: Echinodermata; class: Crinoidea; **Location:** waterBody: Pacific; stateProvince: Clarion Clipperton Zone; locality: UK Seabed Resources Ltd exploration claim UK-1; verbatimLocality: UK-1 Stratum A; maximumDepthInMeters: 4108; locationRemarks: RV Melville Cruise MV1313; decimalLatitude: 13.849883333333; decimalLongitude: -116.64495; geodeticDatum: WGS84; **Identification:** identifiedBy: Adrian Glover, Helena Wiklund, Thomas Dahlgren; dateIdentified: 2015-06-01; identificationRemarks: identified by DNA and morphology; **Event:** samplingProtocol: USNEL Box Core; eventDate: 2013-10-09; eventTime: 17:34; habitat: Abyssal plain; fieldNumber: BC04; fieldNotes: Collected from polymetallic nodule found in benthic sediment; **Record Level:** language: en; institutionCode: NHMUK; collectionCode: ZOO; datasetName: ABYSSLINE; basisOfRecord: PreservedSpecimen

#### Description

**Figure 15.**
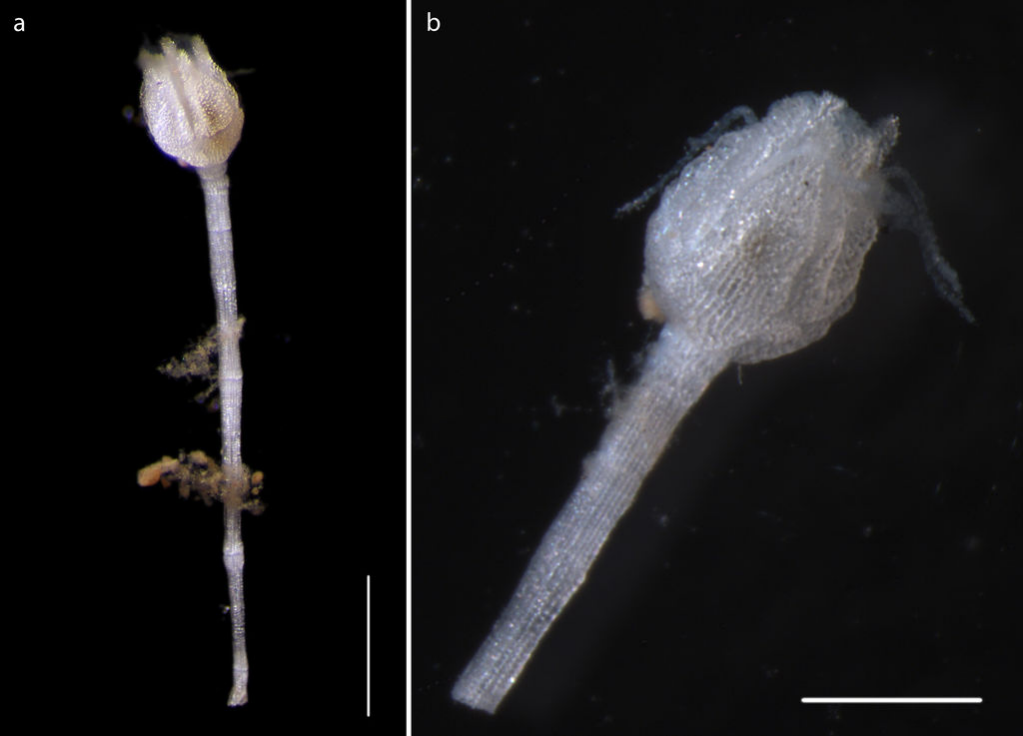
Crinoidea sp. Specimen NHM_056. (a) Specimen after removal from polymetallic nodule. (b) Detail of crown, calyx, pinnules and arms as present after DNA extraction from stalk. Scale bars (a) 1mm, (b) 0.5mm. Image attribution Glover, Dahlgren & Wiklund 2015

Specimen including stalk and crown, calyx with proximal arms only, 5mm in total length. Calyx 0.62mm in width, including proximal arms 0.86mm in length. Distally, pinnules observed on arms arising laterally from arms (Fig. 15).

Genetic data for this taxa with new GenBank accession numbers are provided in Table 2

#### Diagnosis

Forms a unique monophyletic clade distinct from other AB01 specimens. No genetic matches on GenBank or Barcode of Life Database.

#### Ecology

Found on polymetallic nodule.

### Crinoidea sp. 'NHM_300'

#### Materials

- **Type status:** Other material. **Occurrence:** catalogNumber: 2866f91e-b99e-4703-a9d3-fe1876df1da1; recordNumber: NHM_300; recordedBy: Adrian Glover, Helena Wiklund, Thomas Dahlgren, Maggie Georgieva; individualCount: 1; preparations: tissue and DNA voucher stored in 80% non-denatured ethanol aqueous solution; otherCatalogNumbers: 5023510; associatedSequences: http://www.ncbi.nlm.nih.gov/nuccore/KU519549 | KU519517 | KU519534; **Taxon:** taxonConceptID: Crinoidea sp. (NHM_300); scientificName: Crinoidea; kingdom: Animalia; phylum: Echinodermata; class: Crinoidea; **Location:** waterBody: Pacific; stateProvince: Clarion Clipperton Zone; locality: UK Seabed Resources Ltd exploration claim UK-1; verbatimLocality: UK-1 Stratum A; maximumDepthInMeters: 4076; locationRemarks: RV Melville Cruise MV1313; decimalLatitude: 13.755833333333; decimalLongitude: -116.48666666667; geodeticDatum: WGS84; **Identification:** identifiedBy: Adrian Glover, Helena Wiklund, Thomas Dahlgren; dateIdentified: 2015-06-01; identificationRemarks: identified by DNA and morphology; **Event:** samplingProtocol: Brenke Epibenthic Sledge; eventDate: 2013-10-17; eventTime: 01:50; habitat: Abyssal plain; fieldNumber: EB04; fieldNotes: Collected from epi net (on the epibenthic sledge); **Record Level:** language: en; institutionCode: NHMUK; collectionCode: ZOO; datasetName: ABYSSLINE; basisOfRecord: PreservedSpecimen

#### Description

**Figure 16.**
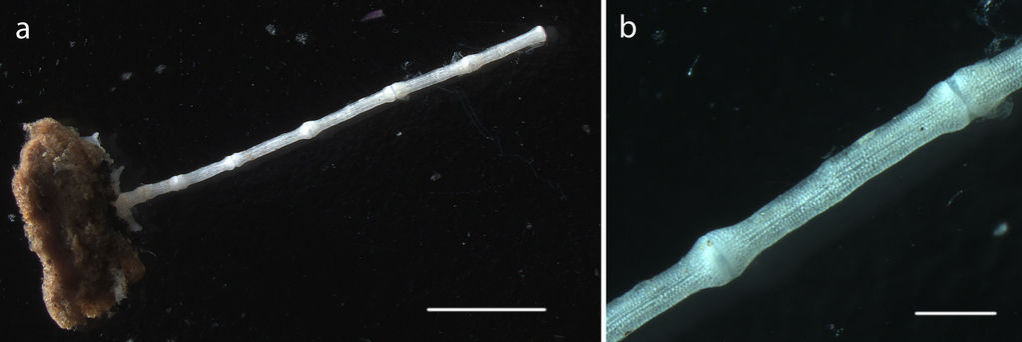
Crinoidea sp. Specimen NHM_300 (a) Specimen found and imaged during shipboard live-sorting. (b) Detail of columnals (crown absent). Scale bars (a) 2mm, (b) 0.5mm. Image attribution Glover, Dahlgren & Wiklund 2015

Specimen lacks calyx, crown, arms. Stalk 6.5mm in length, attached to nodule fragment. Columnals 1.6mm in length, 0.28mm in width (Fig. 16).

Genetic data for this taxa with new GenBank accession numbers are provided in Table 2

#### Diagnosis

Forms a unique monophyletic clade distinct from other AB01 specimens. No genetic matches on GenBank or Barcode of Life Database. Lacks crown and calyx.

#### Ecology

Found on polymetallic nodule.

### Benthodytes sanguinolenta

Théel, 1882

#### Materials

- **Type status:** Other material. **Occurrence:** catalogNumber: d0062182-89dc-4deb-b746-688289783b5f; recordNumber: NHM_216; recordedBy: Adrian Glover, Helena Wiklund, Thomas Dahlgren, Maggie Georgieva; individualCount: 1; preparations: tissue and DNA voucher stored in 80% non-denatured ethanol aqueous solution; otherCatalogNumbers: 5023498; associatedSequences: http://www.ncbi.nlm.nih.gov/nuccore/KU519546 | KU519513; **Taxon:** taxonConceptID: Benthodytes cf. sanguinolenta; scientificName: Benthodytes sanguinolenta; kingdom: Animalia; phylum: Echinodermata; class: Holothuroidea; order: Elasipodida; family: Psychropotidae; genus: Benthodytes; scientificNameAuthorship: Théel, 1882; **Location:** waterBody: Pacific; stateProvince: Clarion Clipperton Zone; locality: UK Seabed Resources Ltd exploration claim UK-1; verbatimLocality: UK-1 Stratum A; maximumDepthInMeters: 4063; locationRemarks: RV Melville Cruise MV1313; decimalLatitude: 13.9629; decimalLongitude: -116.5513; geodeticDatum: WGS84; **Identification:** identifiedBy: Diva Amon, David Pawson, Adrian Glover, Helena Wiklund, Thomas Dahlgren; dateIdentified: 2015-06-01; identificationRemarks: identified by DNA and morphology; identificationQualifier: cf; **Event:** samplingProtocol: Remotely Operated Vehicle; eventDate: 2013-10-15; eventTime: 23:15; habitat: Abyssal plain; fieldNumber: RV03; **Record Level:** language: en; institutionCode: NHMUK; collectionCode: ZOO; datasetName: ABYSSLINE; basisOfRecord: PreservedSpecimen

#### Description

**Figure 17.**
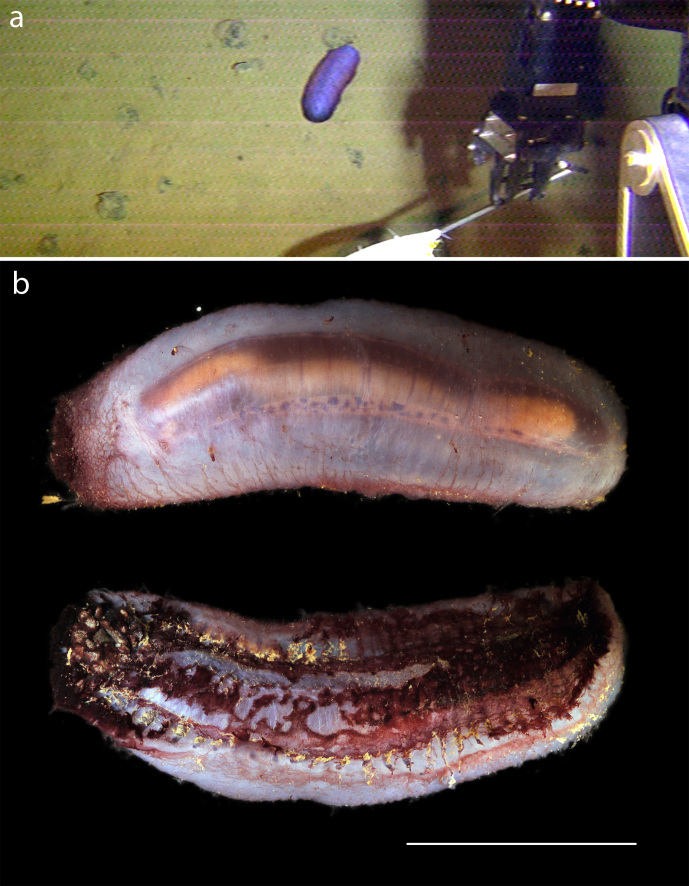
Benthodytes cf. sanguinolenta Théel, 1882. Specimen NHM_216. (a) Specimen NHM_216 *in situ* on seafloor shortly before collection by ROV manipulator arm, (b) Live specimen photographed immediately after recovery from the ROV biobox, upper (dorsal view), lower (ventral view). Scale bar 5cm. Image attribution (a) Smith & Amon 2013, (b) Glover, Dahlgren & Wiklund, 2015.

Morphologically agrees with either *Benthodytes sanguinolenta* or *Benthodytes typica* both from Théel 1882 (Fig. 17).

Genetic data for this taxa with new GenBank accession numbers are provided in Table 2

#### Diagnosis

Forms a unique monophyletic clade distinct from other AB01 specimens and no match (16S) to any GenBank or BOLD databases. Morphologically consistent with *Benthodytes sanguinolenta* or *B. typica*. The type locality of *B. sanguinolenta* is in the Pacific ocean (34°7'S; 73°56'W, 4000m depth) while type locality of *B. typica* is Atlantic (35°47'N; 8°23'W, 2000m depth) (Théel 1882). We assign the tentative name Benthodytes cf. sanguinolent to this material until we have a better understanding of genetic variation within the species *B. sanguinolenta* and *B. typica* including data from the type localities.

#### Ecology

Observed moving on the seabed amongst polymetallic nodules.

### Psychropotes semperiana

Théel, 1882

#### Materials

- **Type status:** Other material. **Occurrence:** catalogNumber: 38c16bec-7bf9-4c2b-b862-5da460ba6c0c; recordNumber: NHM_220; recordedBy: Adrian Glover, Helena Wiklund, Thomas Dahlgren, Maggie Georgieva; individualCount: 1; preparations: tissue and DNA voucher stored in 80% non-denatured ethanol aqueous solution; otherCatalogNumbers: 5023502; associatedSequences: http://www.ncbi.nlm.nih.gov/nuccore/KU519526; **Taxon:** taxonConceptID: Psychropotes cf. semperiana; scientificName: Psychropotes semperiana; kingdom: Animalia; phylum: Echinodermata; class: Holothuroidea; order: Elasipodida; family: Psychropotidae; genus: Psychropotes; scientificNameAuthorship: Théel, 1882; **Location:** waterBody: Pacific; stateProvince: Clarion Clipperton Zone; locality: UK Seabed Resources Ltd exploration claim UK-1; verbatimLocality: UK-1 Stratum A; maximumDepthInMeters: 4062; locationRemarks: RV Melville Cruise MV1313; decimalLatitude: 13.962791666667; decimalLongitude: -116.55092666667; geodeticDatum: WGS84; **Identification:** identifiedBy: Diva Amon, David Pawson, Adrian Glover, Helena Wiklund, Thomas Dahlgren; dateIdentified: 2015-06-01; identificationRemarks: identified by DNA and morphology; identificationQualifier: cf; **Event:** samplingProtocol: Remotely Operated Vehicle; eventDate: 2013-10-15; eventTime: 22:40; habitat: Abyssal plain; fieldNumber: RV03; **Record Level:** language: en; institutionCode: NHMUK; collectionCode: ZOO; datasetName: ABYSSLINE; basisOfRecord: PreservedSpecimen

#### Description

**Figure 18.**
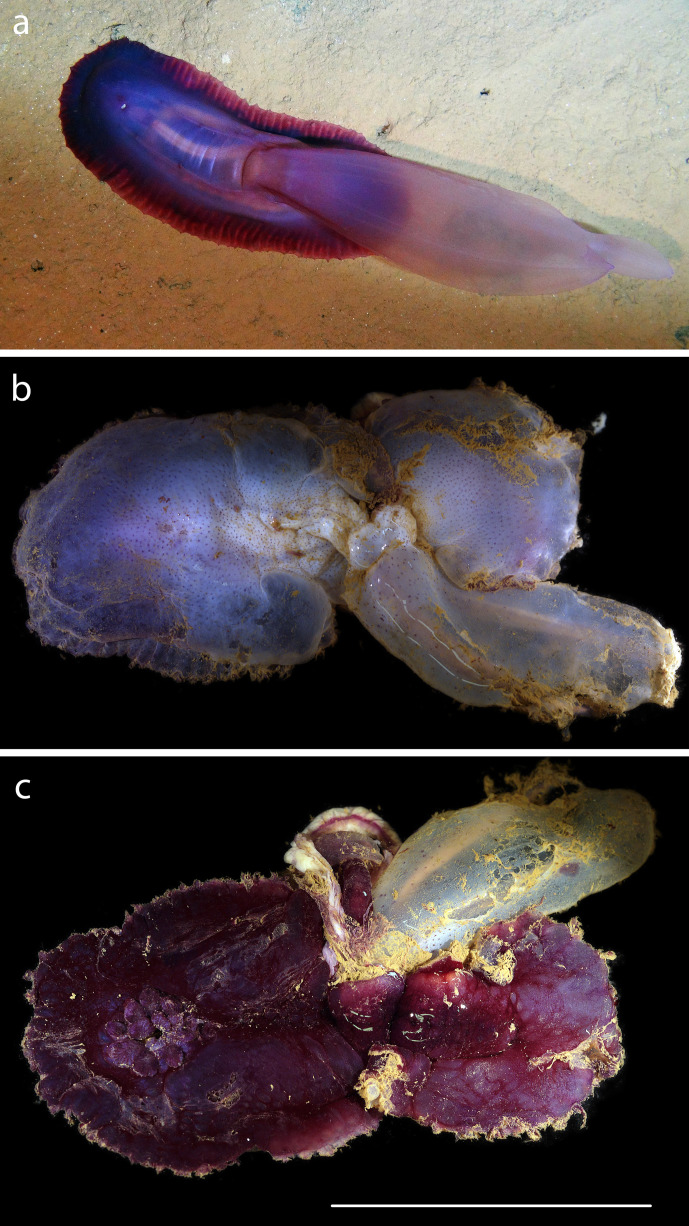
*Psychropotes cf. semperiana* Théel, 1882. Specimen NHM_220. (a) Live specimen photographed in-situ on the seafloor. (b) Same specimen dorsal view after recovery by ROV imaged underwater in cold-water tank. (c) Ventral view. Scale bars (c) 10cm. Image attribution (a) Smith & Amon 2013, (b) Glover, Dahlgren & Wiklund, 2015.

Distinctive large holothurian with sail, close morphological match to *Psychropotes semperiana* from Théel 1882​ (Fig. 18).

Genetic data for this taxa with new GenBank accession numbers are provided in Table 2

#### Diagnosis

Forms a unique monophyletic clade distinct from other AB01 specimens and no match to any GenBank or BOLD databases. Morphologically consistent with *Psychropotes semperiana* Théel, 1882. The type locality of *Psychropotes semperiana* is Atlantic (5°48'N; 14°20'W, 4500m depth) and we use the tentative name *Psychropotes cf. semperiana* for this material until we have a better understanding of genetic variation within the species including data from the type locality.

#### Ecology

Observed moving on the seabed amongst polymetallic nodules.

### Amphioplus daleus

Lyman, 1879

#### Materials

- **Type status:** Other material. **Occurrence:** catalogNumber: 72db478a-ea4f-4f3e-be08-95ec9fb4d20e; recordNumber: NHM_094; recordedBy: Adrian Glover, Helena Wiklund, Thomas Dahlgren, Maggie Georgieva; individualCount: 1; preparations: tissue and DNA voucher stored in 80% non-denatured ethanol aqueous solution; otherCatalogNumbers: 5023485; associatedSequences: http://www.ncbi.nlm.nih.gov/nuccore/KU519544; **Taxon:** taxonConceptID: Amphioplus cf. daleus; scientificName: Amphioplus (Unioplus) daleus; kingdom: Animalia; phylum: Echinodermata; class: Ophiuroidea; order: Ophiurina; family: Amphiuridae; genus: Amphioplus; subgenus: Amphioplus (Unioplus); scientificNameAuthorship: Lyman, 1879; **Location:** waterBody: Pacific; stateProvince: Clarion Clipperton Zone; locality: UK Seabed Resources Ltd exploration claim UK-1; verbatimLocality: UK-1 Stratum A; maximumDepthInMeters: 4081; locationRemarks: RV Melville Cruise MV1313; decimalLatitude: 13.79335; decimalLongitude: -116.70308333333; geodeticDatum: WGS84; **Identification:** identifiedBy: Diva Amon, Tim O'Hara, Adrian Glover, Helena Wiklund, Thomas Dahlgren; dateIdentified: 2015-06-01; identificationRemarks: identified by DNA and morphology; identificationQualifier: cf; **Event:** samplingProtocol: USNEL Box Core; eventDate: 2013-10-11; eventTime: 12:30; habitat: Abyssal plain; fieldNumber: BC05; fieldNotes: Collected from 0-2 cm layer of box core using a 300 micron sieve; **Record Level:** language: en; institutionCode: NHMUK; collectionCode: ZOO; datasetName: ABYSSLINE; basisOfRecord: PreservedSpecimen
- **Type status:** Other material. **Occurrence:** catalogNumber: 15e6ddc7-3ca7-453c-bba5-f84888716505; recordNumber: NHM_447; recordedBy: Adrian Glover, Helena Wiklund, Thomas Dahlgren, Maggie Georgieva; individualCount: 1; preparations: tissue and DNA voucher stored in 80% non-denatured ethanol aqueous solution; otherCatalogNumbers: 5023529; associatedSequences: http://www.ncbi.nlm.nih.gov/nuccore/KU519545 | KU519511 | KU519529; **Taxon:** taxonConceptID: Amphioplus cf. daleus; scientificName: Amphioplus (Unioplus) daleus; kingdom: Animalia; phylum: Echinodermata; class: Ophiuroidea; order: Ophiurina; genus: Amphioplus; subgenus: Amphioplus (Unioplus); scientificNameAuthorship: Lyman, 1879; **Location:** waterBody: Pacific; stateProvince: Clarion Clipperton Zone; locality: UK Seabed Resources Ltd exploration claim UK-1; verbatimLocality: UK-1 Stratum A; maximumDepthInMeters: 4053; locationRemarks: RV Melville Cruise MV1313; decimalLatitude: 13.86335; decimalLongitude: -116.54665; geodeticDatum: WGS84; **Identification:** identifiedBy: Diva Amon, Tim O'Hara, Adrian Glover, Helena Wiklund, Thomas Dahlgren; dateIdentified: 2015-06-01; identificationRemarks: identified by DNA and morphology; identificationQualifier: cf; **Event:** samplingProtocol: Bowers & Connelly Megacore; eventDate: 2013-10-21; eventTime: 08:48; habitat: Abyssal plain; fieldNumber: MC10; **Record Level:** language: en; institutionCode: NHMUK; collectionCode: ZOO; datasetName: ABYSSLINE; basisOfRecord: PreservedSpecimen

#### Description

**Figure 19.**
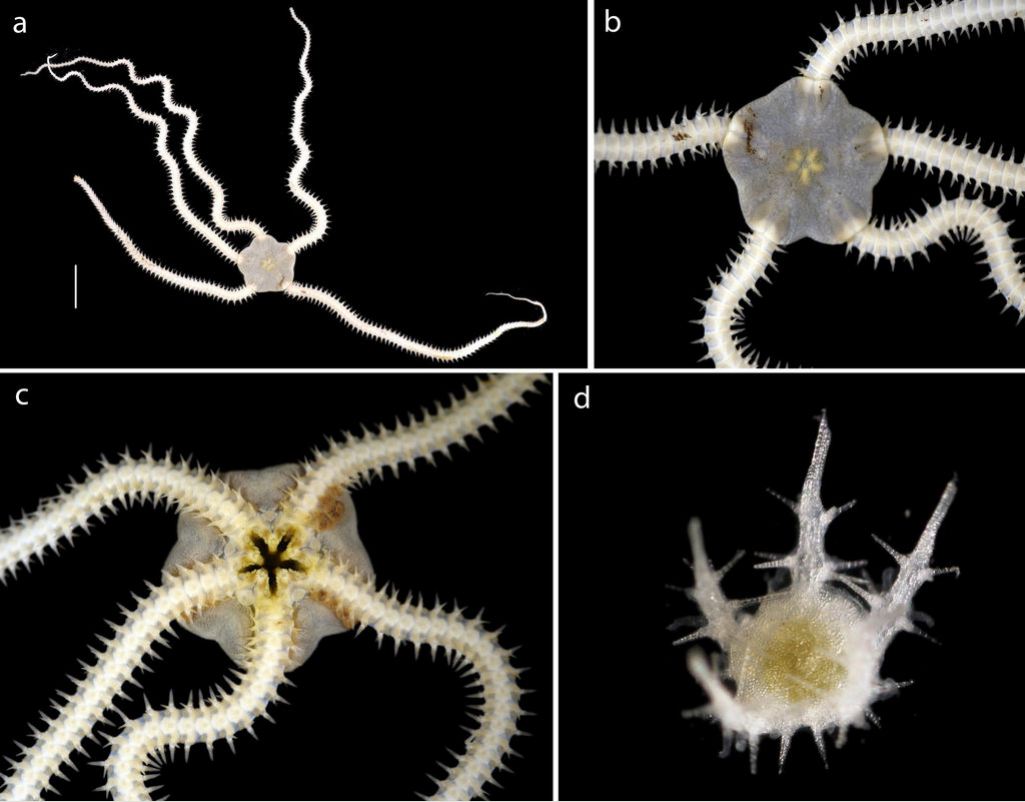
Amphioplus cf. daleus Lyman, 1879. (a) Live specimen NHM_447 imaged dorsal side. (b). Dorsal surface detail. (c). Ventral surface detail. (d) Juvenile, confirmed by DNA data, NHM_094. Scale bars (a) 10mm. Image attribution Glover, Dahlgren & Wiklund, 2015.

Voucher material recovered from megacore sample, specimen with disc of 1cm diameter (Fig. 19). Additional material including juveniles recovered from box core and epibenthic sledge. Agrees with Amphioplus (Unioplus) daleus as detailed in (Lyman 1879).

Genetic data for this taxa with new GenBank accession numbers are provided in Table 2

#### Diagnosis

Forms a unique monophyletic clade distinct from other AB01 specimens. Morphologically consistent with Amphioplus (Unioplus) daleus Lyman 1879. No genetic data for this species yet on GenBank. The type locality of *A. daleus* is Atlantic (36°44’S; 46°16’W; 4800m depth) and we use the tentative name Amphioplus cf. daleus for this material until we have a better understanding of genetic variation within the species including data from the type locality.

#### Ecology

Recovered from a range of sampling gears, NHM_447 recovered alive in top of multiple core tube.

### Ophiomusium glabrum

Lütken & Mortensen, 1899

#### Materials

- **Type status:** Other material. **Occurrence:** catalogNumber: c1c4d8f3-6cd5-439f-a546-943b5e2e8d8f; recordNumber: NHM_009; recordedBy: Adrian Glover, Helena Wiklund, Thomas Dahlgren, Maggie Georgieva; individualCount: 1; preparations: tissue and DNA voucher stored in 80% non-denatured ethanol aqueous solution; otherCatalogNumbers: 5023473; associatedSequences: http://www.ncbi.nlm.nih.gov/nuccore/KU519552; **Taxon:** taxonConceptID: Ophiomusium cf. glabrum; scientificName: Ophiomusium glabrum; kingdom: Animalia; phylum: Echinodermata; class: Ophiuroidea; order: Ophiurina; family: Ophiolepididae; genus: Ophiomusium; scientificNameAuthorship: Lütken & Mortensen, 1899; **Location:** waterBody: Pacific; stateProvince: Clarion Clipperton Zone; locality: UK Seabed Resources Ltd exploration claim UK-1; verbatimLocality: UK-1 Stratum A; maximumDepthInMeters: 4171; locationRemarks: RV Melville Cruise MV1313; decimalLatitude: 13.881666666667; decimalLongitude: -116.46666666667; geodeticDatum: WGS84; **Identification:** identifiedBy: Diva Amon, Tim O'Hara, Adrian Glover, Helena Wiklund, Thomas Dahlgren; dateIdentified: 2015-06-01; identificationRemarks: identified by DNA and morphology; identificationQualifier: cf; **Event:** samplingProtocol: USNEL Box Core; eventDate: 2013-10-08; eventTime: 17:15; habitat: Abyssal plain; fieldNumber: BC03; fieldNotes: Collected from 0-2 cm layer of box core using a 300 micron sieve; **Record Level:** language: en; institutionCode: NHMUK; collectionCode: ZOO; datasetName: ABYSSLINE; basisOfRecord: PreservedSpecimen
- **Type status:** Other material. **Occurrence:** catalogNumber: 4d6f6aaf-93fd-4629-b224-2ce8dd3320f6; recordNumber: NHM_124; recordedBy: Adrian Glover, Helena Wiklund, Thomas Dahlgren, Maggie Georgieva; individualCount: 1; preparations: tissue and DNA voucher stored in 80% non-denatured ethanol aqueous solution; otherCatalogNumbers: 5023489; associatedSequences: http://www.ncbi.nlm.nih.gov/nuccore/KU519553; **Taxon:** taxonConceptID: Ophiomusium cf. glabrum; scientificName: Ophiomusium glabrum; kingdom: Animalia; phylum: Echinodermata; class: Ophiuroidea; order: Ophiurina; family: Ophiolepididae; genus: Ophiomusium; scientificNameAuthorship: Lütken & Mortensen, 1899; **Location:** waterBody: Pacific; stateProvince: Clarion Clipperton Zone; locality: UK Seabed Resources Ltd exploration claim UK-1; verbatimLocality: UK-1 Stratum A; maximumDepthInMeters: 4080; locationRemarks: RV Melville Cruise MV1313; decimalLatitude: 13.758333333333; decimalLongitude: -116.69851666667; geodeticDatum: WGS84; **Identification:** identifiedBy: Diva Amon, Tim O'Hara, Adrian Glover, Helena Wiklund, Thomas Dahlgren; dateIdentified: 2015-06-01; identificationRemarks: identified by DNA and morphology; identificationQualifier: cf; **Event:** samplingProtocol: Brenke Epibenthic Sledge; eventDate: 2013-10-11; eventTime: 10:32; habitat: Abyssal plain; fieldNumber: EB02; fieldNotes: Collected from epi net (on the epibenthic sledge); **Record Level:** language: en; institutionCode: NHMUK; collectionCode: ZOO; datasetName: ABYSSLINE; basisOfRecord: PreservedSpecimen
- **Type status:** Other material. **Occurrence:** catalogNumber: 2ed865af-1605-4d78-8fd8-9c7659781854; recordNumber: NHM_256; recordedBy: Adrian Glover, Helena Wiklund, Thomas Dahlgren, Maggie Georgieva; individualCount: 1; preparations: tissue and DNA voucher stored in 80% non-denatured ethanol aqueous solution; otherCatalogNumbers: 5023507; associatedSequences: http://www.ncbi.nlm.nih.gov/nuccore/KU519554; **Taxon:** taxonConceptID: Ophiomusium cf. glabrum; scientificName: Ophiomusium glabrum; kingdom: Animalia; phylum: Echinodermata; class: Ophiuroidea; order: Ophiurina; family: Ophiolepididae; genus: Ophiomusium; scientificNameAuthorship: Lütken & Mortensen, 1899; **Location:** waterBody: Pacific; stateProvince: Clarion Clipperton Zone; locality: UK Seabed Resources Ltd exploration claim UK-1; verbatimLocality: UK-1 Stratum A; maximumDepthInMeters: 4076; locationRemarks: RV Melville Cruise MV1313; decimalLatitude: 13.755833333333; decimalLongitude: -116.48666666667; geodeticDatum: WGS84; **Identification:** identifiedBy: Diva Amon, Chris Mah, Adrian Glover, Helena Wiklund, Thomas Dahlgren; dateIdentified: 2015-06-01; identificationRemarks: identified by DNA and morphology; identificationQualifier: cf; **Event:** samplingProtocol: Brenke Epibenthic Sledge; eventDate: 2013-10-17; eventTime: 01:50; habitat: Abyssal plain; fieldNumber: EB04; fieldNotes: Collected from epi net (on the epibenthic sledge); **Record Level:** language: en; institutionCode: NHMUK; collectionCode: ZOO; datasetName: ABYSSLINE; basisOfRecord: PreservedSpecimen
- **Type status:** Other material. **Occurrence:** catalogNumber: 11948cb9-654f-4519-a654-f134380093ea; recordNumber: NHM_329; recordedBy: Adrian Glover, Helena Wiklund, Thomas Dahlgren, Maggie Georgieva; individualCount: 1; preparations: tissue and DNA voucher stored in 80% non-denatured ethanol aqueous solution; otherCatalogNumbers: 5023512; associatedSequences: http://www.ncbi.nlm.nih.gov/nuccore/KU519555 | KU519519 | KU519536; **Taxon:** taxonConceptID: Ophiomusium cf. glabrum; scientificName: Ophiomusium glabrum; kingdom: Animalia; phylum: Echinodermata; class: Ophiuroidea; order: Ophiurina; family: Ophiolepididae; genus: Ophiomusium; scientificNameAuthorship: Lütken & Mortensen, 1899; **Location:** waterBody: Pacific; stateProvince: Clarion Clipperton Zone; locality: UK Seabed Resources Ltd exploration claim UK-1; verbatimLocality: UK-1 Stratum A; maximumDepthInMeters: 4075; locationRemarks: RV Melville Cruise MV1313; decimalLatitude: 13.76085; decimalLongitude: -116.4653; geodeticDatum: WGS84; **Identification:** identifiedBy: Diva Amon, Tim O'Hara, Adrian Glover, Helena Wiklund, Thomas Dahlgren; dateIdentified: 2015-06-01; identificationRemarks: identified by DNA and morphology; identificationQualifier: cf; **Event:** samplingProtocol: Remotely Operated Vehicle; eventDate: 2013-10-17; eventTime: 19:06; habitat: Abyssal plain; fieldNumber: RV05; **Record Level:** language: en; institutionCode: NHMUK; collectionCode: ZOO; datasetName: ABYSSLINE; basisOfRecord: PreservedSpecimen
- **Type status:** Other material. **Occurrence:** catalogNumber: 292bd655-83d6-440f-9668-82dfa4185b04; recordNumber: NHM_335; recordedBy: Adrian Glover, Helena Wiklund, Thomas Dahlgren, Maggie Georgieva; individualCount: 1; preparations: tissue and DNA voucher stored in 80% non-denatured ethanol aqueous solution; otherCatalogNumbers: 5023513; associatedSequences: http://www.ncbi.nlm.nih.gov/nuccore/KU519556; **Taxon:** taxonConceptID: Ophiomusium cf. glabrum; scientificName: Ophiomusium glabrum; kingdom: Animalia; phylum: Echinodermata; class: Ophiuroidea; order: Ophiurina; family: Ophiolepididae; genus: Ophiomusium; scientificNameAuthorship: Lütken & Mortensen, 1899; **Location:** waterBody: Pacific; stateProvince: Clarion Clipperton Zone; locality: UK Seabed Resources Ltd exploration claim UK-1; verbatimLocality: UK-1 Stratum A; maximumDepthInMeters: 4075; locationRemarks: RV Melville Cruise MV1313; decimalLatitude: 13.76085; decimalLongitude: -116.4653; geodeticDatum: WGS84; **Identification:** identifiedBy: Diva Amon, Tim O'Hara, Adrian Glover, Helena Wiklund, Thomas Dahlgren; dateIdentified: 2015-06-01; identificationRemarks: identified by DNA and morphology; identificationQualifier: cf; **Event:** samplingProtocol: Remotely Operated Vehicle; eventDate: 2013-10-17; eventTime: 19:06; habitat: Abyssal plain; fieldNumber: RV05; **Record Level:** language: en; institutionCode: NHMUK; collectionCode: ZOO; datasetName: ABYSSLINE; basisOfRecord: PreservedSpecimen
- **Type status:** Other material. **Occurrence:** catalogNumber: 68072fc9-3e84-4202-8e97-6c9c0c5fc83d; recordNumber: NHM_415; recordedBy: Adrian Glover, Helena Wiklund, Thomas Dahlgren, Maggie Georgieva; individualCount: 1; preparations: tissue and DNA voucher stored in 80% non-denatured ethanol aqueous solution; otherCatalogNumbers: 5023522; associatedSequences: http://www.ncbi.nlm.nih.gov/nuccore/KU519557; **Taxon:** taxonConceptID: Ophiomusium cf. glabrum; scientificName: Ophiomusium glabrum; kingdom: Animalia; phylum: Echinodermata; class: Ophiuroidea; order: Ophiurina; family: Ophiolepididae; genus: Ophiomusium; scientificNameAuthorship: Lütken & Mortensen, 1899; **Location:** waterBody: Pacific; stateProvince: Clarion Clipperton Zone; locality: UK Seabed Resources Ltd exploration claim UK-1; verbatimLocality: UK-1 Stratum A; maximumDepthInMeters: 4050; locationRemarks: RV Melville Cruise MV1313; decimalLatitude: 13.863666666667; decimalLongitude: -116.54431666667; geodeticDatum: WGS84; **Identification:** identifiedBy: Diva Amon, Tim O'Hara, Adrian Glover, Helena Wiklund, Thomas Dahlgren; dateIdentified: 2015-06-01; identificationRemarks: identified by DNA and morphology; identificationQualifier: cf; **Event:** samplingProtocol: Remotely Operated Vehicle; eventDate: 2013-10-20; eventTime: 10:32; habitat: Abyssal plain; fieldNumber: RV06; **Record Level:** language: en; institutionCode: NHMUK; collectionCode: ZOO; datasetName: ABYSSLINE; basisOfRecord: PreservedSpecimen
- **Type status:** Other material. **Occurrence:** catalogNumber: 5ad996fe-134a-4625-a404-9d0cdae435d4; recordNumber: NHM_452; recordedBy: Adrian Glover, Helena Wiklund, Thomas Dahlgren, Maggie Georgieva; individualCount: 1; preparations: tissue and DNA voucher stored in 80% non-denatured ethanol aqueous solution; otherCatalogNumbers: 5023534; associatedSequences: http://www.ncbi.nlm.nih.gov/nuccore/KU519558; **Taxon:** taxonConceptID: Ophiomusium cf. glabrum; scientificName: Ophiomusium glabrum; kingdom: Animalia; phylum: Echinodermata; class: Ophiuroidea; order: Ophiurina; family: Ophiolepididae; genus: Ophiomusium; scientificNameAuthorship: Lütken & Mortensen, 1899; **Location:** waterBody: Pacific; stateProvince: Clarion Clipperton Zone; locality: UK Seabed Resources Ltd exploration claim UK-1; verbatimLocality: UK-1 Stratum A; maximumDepthInMeters: 4050; locationRemarks: RV Melville Cruise MV1313; decimalLatitude: 13.863666666667; decimalLongitude: -116.54431666667; geodeticDatum: WGS84; **Identification:** identifiedBy: Diva Amon, Tim O'Hara, Adrian Glover, Helena Wiklund, Thomas Dahlgren; dateIdentified: 2015-06-01; identificationRemarks: identified by DNA and morphology; identificationQualifier: cf; **Event:** samplingProtocol: Remotely Operated Vehicle; eventDate: 2013-10-20; eventTime: 10:32; habitat: Abyssal plain; fieldNumber: RV06; **Record Level:** language: en; institutionCode: NHMUK; collectionCode: ZOO; datasetName: ABYSSLINE; basisOfRecord: PreservedSpecimen

#### Description

**Figure 20.**
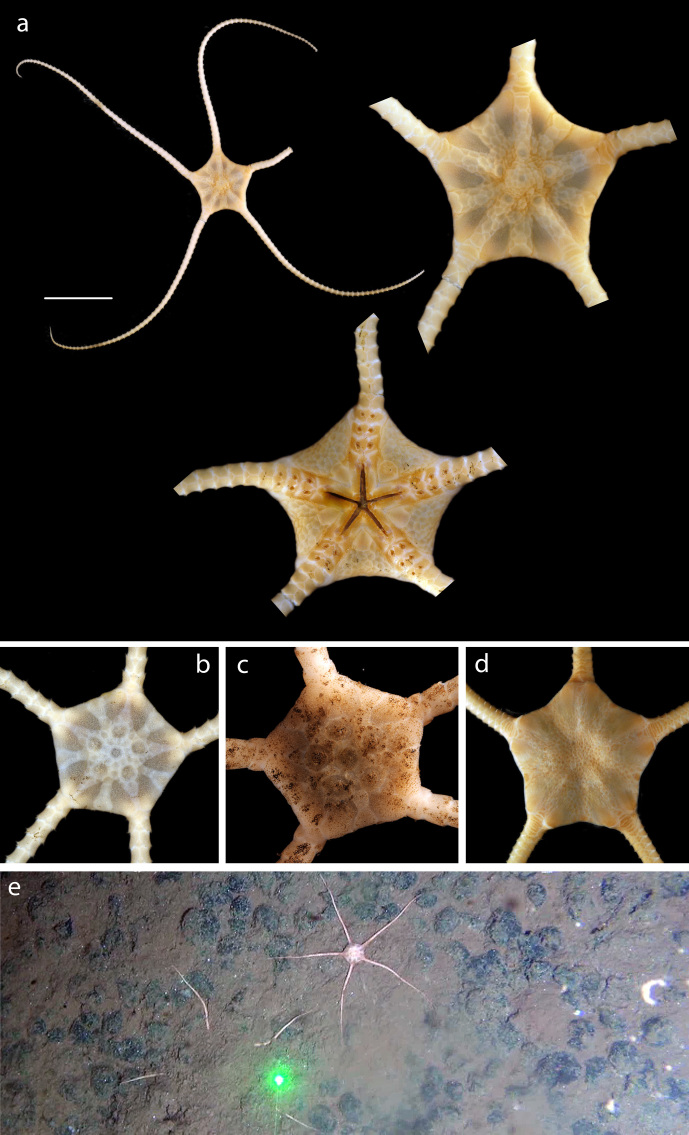
Ophiomusium cf. glabrum Lütken and Mortensen, 1899. (a) Voucher material Specimen NHM_329 with insets showing detail of dorsal and ventral surface of disc. (b) NHM_124. (c) NHM_256. (d) NHM_338. (e) Unsampled specimens of suspected O. cf glabrum imaged during ROV surveys, showing 1 specimen on sediment surface and 1 specimen partially buried in sediment (green dot is a laser scale marker, cropped here). All voucher material specimens and designations confirmed with DNA data. Scale bars (a) 20mm, (g) 2mm. Image attribution (a-d) Glover, Dahlgren & Wiklund 2015 (e) Smith & Amon 2013.

Voucher material, NHM_329, disc approx 20mm in diameter. Additional voucher material (12 specimens) ranges in size from 2mm to 20mm in diameter (Fig. 20). Range of polymorphs observed characterised by pattern of disc dorsal coloration (Fig. 20). Juveniles observed and identified from DNA data.

Genetic data for this taxa with new GenBank accession numbers are provided in Table 2

#### Diagnosis

Forms a unique monophyletic clade distinct from other AB01 specimens. Morphologically fits Ophiomusium cf. glabrum detailed in (Lütken and Mortensen 1899) but no genetic data available from type locality (47°22'N; 125°48'W, 1604m depth) (National Museum of Natural History, Smithsonian Institution 2015). CO1 sequences of our material is 17% different (K2P distance) from sequences published on Genbank (HM400322-HM400323).

#### Ecology

The most abundant brittle-star in the UK-1 exploration claim survey box UK-1 Stratum A, frequently observed by the ROV on the sediment surface and on nodules.

### Ophiotholia sp. 'NHM_076'

#### Materials

- **Type status:** Other material. **Occurrence:** catalogNumber: bd6fe2ce-b4ae-470e-8bdc-cf28a94c6208; recordNumber: NHM_076; recordedBy: Adrian Glover, Helena Wiklund, Thomas Dahlgren, Maggie Georgieva; individualCount: 1; preparations: tissue and DNA voucher stored in 80% non-denatured ethanol aqueous solution; otherCatalogNumbers: 5023482; associatedSequences: http://www.ncbi.nlm.nih.gov/nuccore/KU519559 | KU519520 | KU519537; **Taxon:** taxonConceptID: Ophiotholia sp. (NHM_076); scientificName: Ophiotholia; kingdom: Animalia; phylum: Echinodermata; class: Ophiuroidea; order: Ophiurina; family: Ophiomycetidae; genus: Ophiotholia; scientificNameAuthorship: Lyman, 1880; **Location:** waterBody: Pacific; stateProvince: Clarion Clipperton Zone; locality: UK Seabed Resources Ltd exploration claim UK-1; verbatimLocality: UK-1 Stratum A; maximumDepthInMeters: 4108; locationRemarks: RV Melville Cruise MV1313; decimalLatitude: 13.849883333333; decimalLongitude: -116.64495; geodeticDatum: WGS84; **Identification:** identifiedBy: Adrian Glover, Helena Wiklund, Thomas Dahlgren; dateIdentified: 2015-06-01; identificationRemarks: identified by DNA and morphology; **Event:** samplingProtocol: USNEL Box Core; eventDate: 2013-10-09; eventTime: 17:34; habitat: Abyssal plain; fieldNumber: BC04; fieldNotes: Collected from 0-2 cm layer of box core using a 300 micron sieve; **Record Level:** language: en; institutionCode: NHMUK; collectionCode: ZOO; datasetName: ABYSSLINE; basisOfRecord: PreservedSpecimen
- **Type status:** Other material. **Occurrence:** catalogNumber: 97d40306-fe6c-4911-8e68-1f9efc3d838f; recordNumber: NHM_078; recordedBy: Adrian Glover, Helena Wiklund, Thomas Dahlgren, Maggie Georgieva; individualCount: 1; preparations: tissue and DNA voucher stored in 80% non-denatured ethanol aqueous solution; otherCatalogNumbers: 5023483; associatedSequences: http://www.ncbi.nlm.nih.gov/nuccore/KU519560; **Taxon:** taxonConceptID: Ophiotholia sp. (NHM_076); scientificName: Ophiotholia; kingdom: Animalia; phylum: Echinodermata; class: Ophiuroidea; order: Ophiurina; family: Ophiomycetidae; genus: Ophiotholia; scientificNameAuthorship: Lyman, 1880; **Location:** waterBody: Pacific; stateProvince: Clarion Clipperton Zone; locality: UK Seabed Resources Ltd exploration claim UK-1; verbatimLocality: UK-1 Stratum A; maximumDepthInMeters: 4108; locationRemarks: RV Melville Cruise MV1313; decimalLatitude: 13.849883333333; decimalLongitude: -116.64495; geodeticDatum: WGS84; **Identification:** identifiedBy: Adrian Glover, Helena Wiklund, Thomas Dahlgren; dateIdentified: 2015-06-01; identificationRemarks: identified by DNA and morphology; **Event:** samplingProtocol: USNEL Box Core; eventDate: 2013-10-09; eventTime: 17:34; habitat: Abyssal plain; fieldNumber: BC04; fieldNotes: Collected from 0-2 cm layer of box core using a 300 micron sieve; **Record Level:** language: en; institutionCode: NHMUK; collectionCode: ZOO; datasetName: ABYSSLINE; basisOfRecord: PreservedSpecimen
- **Type status:** Other material. **Occurrence:** catalogNumber: 479218ae-813b-4736-b3f2-7eec63640ffd; recordNumber: NHM_104; recordedBy: Adrian Glover, Helena Wiklund, Thomas Dahlgren, Maggie Georgieva; individualCount: 1; preparations: tissue and DNA voucher stored in 80% non-denatured ethanol aqueous solution; otherCatalogNumbers: 5023486; associatedSequences: http://www.ncbi.nlm.nih.gov/nuccore/KU519561; **Taxon:** taxonConceptID: Ophiotholia sp. (NHM_076); scientificName: Ophiotholia; kingdom: Animalia; phylum: Echinodermata; class: Ophiuroidea; order: Ophiurina; family: Ophiomycetidae; genus: Ophiotholia; scientificNameAuthorship: Lyman, 1880; **Location:** waterBody: Pacific; stateProvince: Clarion Clipperton Zone; locality: UK Seabed Resources Ltd exploration claim UK-1; verbatimLocality: UK-1 Stratum A; maximumDepthInMeters: 4081; locationRemarks: RV Melville Cruise MV1313; decimalLatitude: 13.79335; decimalLongitude: -116.70308333333; geodeticDatum: WGS84; **Identification:** identifiedBy: Adrian Glover, Helena Wiklund, Thomas Dahlgren; dateIdentified: 2015-06-01; identificationRemarks: identified by DNA and morphology; **Event:** samplingProtocol: USNEL Box Core; eventDate: 2013-10-11; eventTime: 12:30; habitat: Abyssal plain; fieldNumber: BC05; fieldNotes: Collected from 0-2 cm layer of box core using a 300 micron sieve; **Record Level:** language: en; institutionCode: NHMUK; collectionCode: ZOO; datasetName: ABYSSLINE; basisOfRecord: PreservedSpecimen
- **Type status:** Other material. **Occurrence:** catalogNumber: 90e22ace-ef5d-4cb5-a4a5-29fcd55ed660; recordNumber: NHM_119; recordedBy: Adrian Glover, Helena Wiklund, Thomas Dahlgren, Maggie Georgieva; individualCount: 1; preparations: tissue and DNA voucher stored in 80% non-denatured ethanol aqueous solution; otherCatalogNumbers: 5023487; associatedSequences: http://www.ncbi.nlm.nih.gov/nuccore/KU519562; **Taxon:** taxonConceptID: Ophiotholia sp. (NHM_076); scientificName: Ophiotholia; kingdom: Animalia; phylum: Echinodermata; class: Ophiuroidea; order: Ophiurina; family: Ophiomycetidae; genus: Ophiotholia; scientificNameAuthorship: Lyman, 1880; **Location:** waterBody: Pacific; stateProvince: Clarion Clipperton Zone; locality: UK Seabed Resources Ltd exploration claim UK-1; verbatimLocality: UK-1 Stratum A; maximumDepthInMeters: 4081; locationRemarks: RV Melville Cruise MV1313; decimalLatitude: 13.79335; decimalLongitude: -116.70308333333; geodeticDatum: WGS84; **Identification:** identifiedBy: Adrian Glover, Helena Wiklund, Thomas Dahlgren; dateIdentified: 2015-06-01; identificationRemarks: identified by DNA and morphology; **Event:** samplingProtocol: USNEL Box Core; eventDate: 2013-10-11; eventTime: 12:30; habitat: Abyssal plain; fieldNumber: BC05; fieldNotes: Collected from 0-2 cm layer of box core using a 300 micron sieve; **Record Level:** language: en; institutionCode: NHMUK; collectionCode: ZOO; datasetName: ABYSSLINE; basisOfRecord: PreservedSpecimen

#### Description

**Figure 21.**
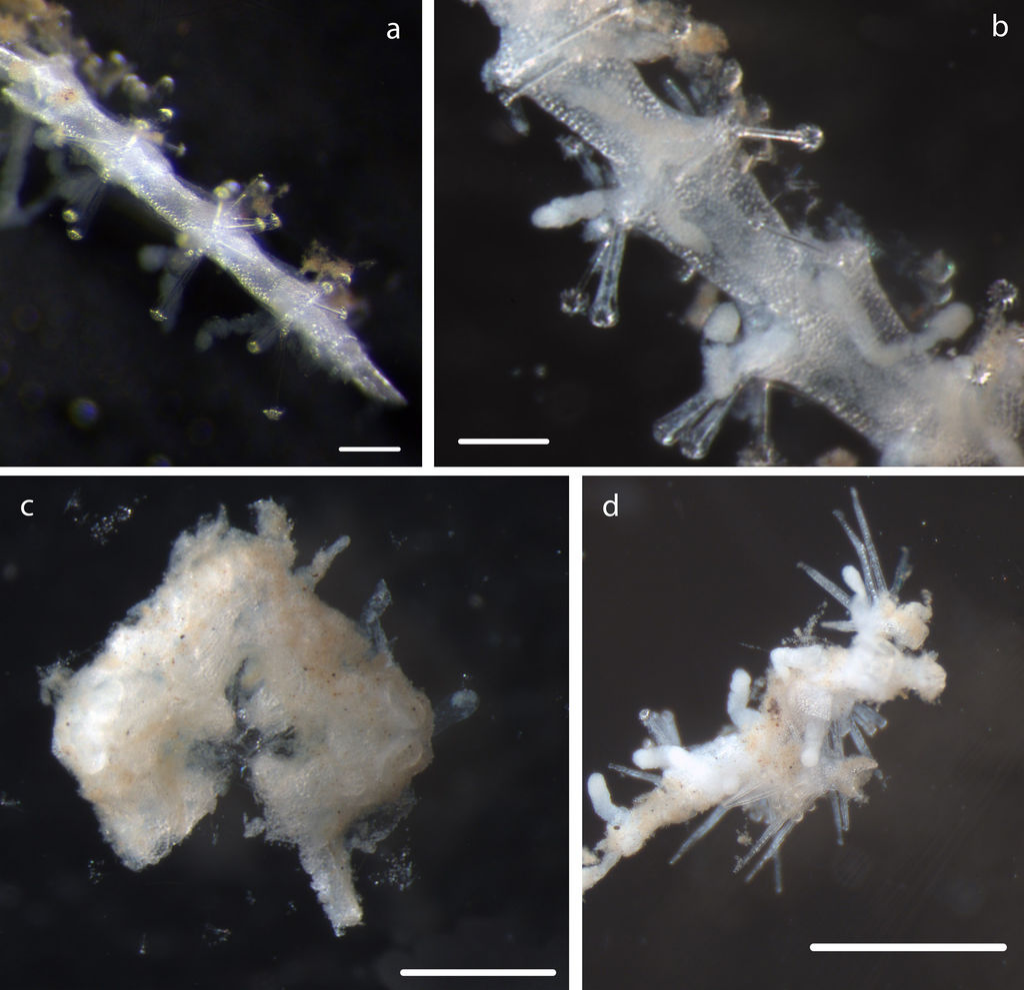
*Ophiotholia* sp. specimen fragments, identified through DNA. (a) NHM_076. (b) NHM_076 detail. (c) NHM_104. (d) NHM_78. Scale bars (a) 300µm, (b) 200µm, (c) 500µm, (d) 1mm. Image attribution Glover, Dahlgren & Wiklund, 2015.

Voucher material, consisting of a series of fragments of arms and one partial disc. All material specimens form a monophyletic clade based on DNA. Arm processes (parasols) suggestive of *Ophiotholia* sp affinity. In NHM_076, arms are 0.31mm wide, with parasol-shaped processes of 0.19mm in length, parasols, 0.048mm in width (Fig. 21).

Genetic data for this taxa with new GenBank accession numbers are provided in Table 2

#### Diagnosis

Forms a unique monophyletic clade distinct from other AB01 specimens. Morphologically perhaps close to *Ophiotholia* but requires further sampling.

#### Ecology

Specimens recovered from two box cores, two specimens from each. Specimens from the same box cores genetically identical, so could be fragments of the same species.

### Ophiuroidea incertae sedis sp. 'NHM_041'

#### Materials

- **Type status:** Other material. **Occurrence:** catalogNumber: 608349ff-5adf-4e1e-8cd7-7e0e41aee222; recordNumber: NHM_041; recordedBy: Adrian Glover, Helena Wiklund, Thomas Dahlgren, Maggie Georgieva; individualCount: 1; preparations: tissue and DNA voucher stored in 80% non-denatured ethanol aqueous solution; otherCatalogNumbers: 5023475; associatedSequences: http://www.ncbi.nlm.nih.gov/nuccore/KU519563 | KU519521 | KU519538; **Taxon:** taxonConceptID: Ophiuroidea incertae sedis sp. (NHM_041); scientificName: Ophiuroidea; kingdom: Animalia; phylum: Echinodermata; class: Ophiuroidea; **Location:** waterBody: Pacific; stateProvince: Clarion Clipperton Zone; locality: UK Seabed Resources Ltd exploration claim UK-1; verbatimLocality: UK-1 Stratum A; maximumDepthInMeters: 4336; locationRemarks: RV Melville Cruise MV1313; decimalLatitude: 13.8372; decimalLongitude: -116.55843333333; geodeticDatum: WGS84; **Identification:** identifiedBy: Adrian Glover, Helena Wiklund, Thomas Dahlgren; dateIdentified: 2015-06-01; identificationRemarks: identified by DNA and morphology; identificationQualifier: incertae sedis; **Event:** samplingProtocol: Brenke Epibenthic Sledge; eventDate: 2013-10-09; eventTime: 10:26; habitat: Abyssal plain; fieldNumber: EB01; fieldNotes: Collected from epi net (on the epibenthic sledge); **Record Level:** language: en; institutionCode: NHMUK; collectionCode: ZOO; datasetName: ABYSSLINE; basisOfRecord: PreservedSpecimen

#### Description

**Figure 22.**
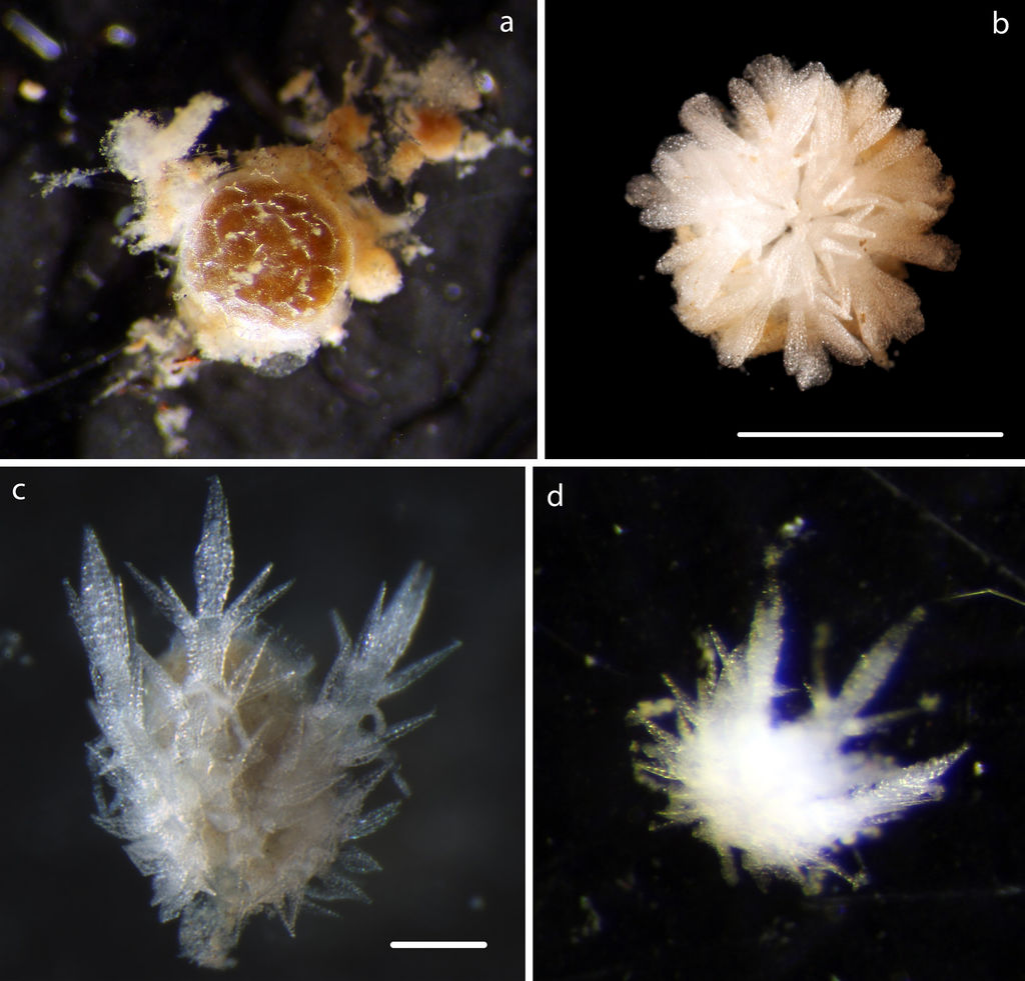
Ophiuroidea incertae sedis, specimen fragments, identified through DNA. (a) Ophiuroidea sp, NHM_072. (b) Ophiuroidea sp, NHM_041. (c) Ophiuroidea sp, NHM_303. (d) Ophiuroidea sp, NHM_371. Scale bars (b) 1mm, (c) 0.2mm. Image attribution Glover, Dahlgren & Wiklund, 2015.

Small disc fragments found in several samples, distinct petal arrangement visible ventrally (Fig. 22).

Genetic data for this taxa with new GenBank accession numbers are provided in Table 2

#### Diagnosis

Forms a unique monophyletic clade distinct from other AB01 specimens. Morphologically not recognisable.

### Ophiuroidea incertae sedis sp. 'NHM_072'

#### Materials

- **Type status:** Other material. **Occurrence:** catalogNumber: 241d094a-568f-4194-997c-fd08f67dcdac; recordNumber: NHM_072; recordedBy: Adrian Glover, Helena Wiklund, Thomas Dahlgren, Maggie Georgieva; individualCount: 1; preparations: tissue and DNA voucher stored in 80% non-denatured ethanol aqueous solution; otherCatalogNumbers: 5023481; associatedSequences: http://www.ncbi.nlm.nih.gov/nuccore/KU519564 | KU519522 | KU519539; **Taxon:** taxonConceptID: Ophiuroidea incertae sedis sp. (NHM_072); scientificName: Ophiuroidea; kingdom: Animalia; phylum: Echinodermata; class: Ophiuroidea; **Location:** waterBody: Pacific; stateProvince: Clarion Clipperton Zone; locality: UK Seabed Resources Ltd exploration claim UK-1; verbatimLocality: UK-1 Stratum A; maximumDepthInMeters: 4108; locationRemarks: RV Melville Cruise MV1313; decimalLatitude: 13.849883333333; decimalLongitude: -116.64495; geodeticDatum: WGS84; **Identification:** identifiedBy: Adrian Glover, Helena Wiklund, Thomas Dahlgren; dateIdentified: 2015-06-01; identificationRemarks: identified by DNA and morphology; identificationQualifier: incertae sedis; **Event:** samplingProtocol: USNEL Box Core; eventDate: 2013-10-09; eventTime: 17:34; habitat: Abyssal plain; fieldNumber: BC04; fieldNotes: Collected from 0-2 cm layer of box core using a 300 micron sieve; **Record Level:** language: en; institutionCode: NHMUK; collectionCode: ZOO; datasetName: ABYSSLINE; basisOfRecord: PreservedSpecimen

#### Description

Small fragment consisting of orange-coloured disc, arms absent or missing (Fig. 22).

Genetic data for this taxa with new GenBank accession numbers are provided in Table 2

#### Diagnosis

Forms a unique monophyletic clade distinct from other AB01 specimens. Morphologically not recognisable.

### Ophiuroidea incertae sedis sp. 'NHM_303'

#### Materials

- **Type status:** Other material. **Occurrence:** catalogNumber: e9f38ce3-5ed5-49f3-8713-c26de2eefd2b; recordNumber: NHM_303; recordedBy: Adrian Glover, Helena Wiklund, Thomas Dahlgren, Maggie Georgieva; individualCount: 1; preparations: tissue and DNA voucher stored in 80% non-denatured ethanol aqueous solution; otherCatalogNumbers: 5023511; associatedSequences: http://www.ncbi.nlm.nih.gov/nuccore/KU519565 | KU519523 | KU519540; **Taxon:** taxonConceptID: Ophiuroidea incertae sedis sp. (NHM_303); scientificName: Ophiuroidea; kingdom: Animalia; phylum: Echinodermata; class: Ophiuroidea; **Location:** waterBody: Pacific; stateProvince: Clarion Clipperton Zone; locality: UK Seabed Resources Ltd exploration claim UK-1; verbatimLocality: UK-1 Stratum A; maximumDepthInMeters: 4110; locationRemarks: RV Melville Cruise MV1313; decimalLatitude: 13.7621; decimalLongitude: -116.46375; geodeticDatum: WGS84; **Identification:** identifiedBy: Adrian Glover, Helena Wiklund, Thomas Dahlgren; dateIdentified: 2015-06-01; identificationRemarks: identified by DNA and morphology; identificationQualifier: incertae sedis; **Event:** samplingProtocol: USNEL Box Core; eventDate: 2013-10-17; eventTime: 13:40; habitat: Abyssal plain; fieldNumber: BC09; fieldNotes: Collected from 0-2 cm layer of box core using a 300 micron sieve; **Record Level:** language: en; institutionCode: NHMUK; collectionCode: ZOO; datasetName: ABYSSLINE; basisOfRecord: PreservedSpecimen
- **Type status:** Other material. **Occurrence:** catalogNumber: 93b0a70d-c74e-4735-b70e-0c6e4c6a36ff; recordNumber: NHM_371; recordedBy: Adrian Glover, Helena Wiklund, Thomas Dahlgren, Maggie Georgieva; individualCount: 1; preparations: tissue and DNA voucher stored in 80% non-denatured ethanol aqueous solution; otherCatalogNumbers: 5023515; associatedSequences: http://www.ncbi.nlm.nih.gov/nuccore/KU519566; **Taxon:** taxonConceptID: Ophiuroidea incertae sedis sp. (NHM_303); scientificName: Ophiuroidea; kingdom: Animalia; phylum: Echinodermata; class: Ophiuroidea; **Location:** waterBody: Pacific; stateProvince: Clarion Clipperton Zone; locality: UK Seabed Resources Ltd exploration claim UK-1; verbatimLocality: UK-1 Stratum A; maximumDepthInMeters: 4182; locationRemarks: RV Melville Cruise MV1313; decimalLatitude: 13.933066666667; decimalLongitude: -116.71628333333; geodeticDatum: WGS84; **Identification:** identifiedBy: Adrian Glover, Helena Wiklund, Thomas Dahlgren; dateIdentified: 2015-06-01; identificationRemarks: identified by DNA and morphology; identificationQualifier: incertae sedis; **Event:** samplingProtocol: Brenke Epibenthic Sledge; eventDate: 2013-10-19; eventTime: 12:16; habitat: Abyssal plain; fieldNumber: EB05; fieldNotes: Collected from epi net (on the epibenthic sledge); **Record Level:** language: en; institutionCode: NHMUK; collectionCode: ZOO; datasetName: ABYSSLINE; basisOfRecord: PreservedSpecimen

#### Description

Small fragments found in several samples, distinct upturned arms and prounonced hump on crest (Fig. 22).

Genetic data for this taxa with new GenBank accession numbers are provided in Table 2

#### Diagnosis

Forms a unique monophyletic clade distinct from other AB01 specimens. Morphologically not recognisable.

### Perlophiura profundissima

Belyaev & Litvinova, 1972

#### Materials

- **Type status:** Other material. **Occurrence:** catalogNumber: f263bc90-6307-462c-9e02-7b87d20e2840; recordNumber: NHM_257; recordedBy: Adrian Glover, Helena Wiklund, Thomas Dahlgren, Maggie Georgieva; individualCount: 1; preparations: tissue and DNA voucher stored in 80% non-denatured ethanol aqueous solution; otherCatalogNumbers: 5023508; associatedSequences: http://www.ncbi.nlm.nih.gov/nuccore/KU519567 | KU519524 | KU519541; **Taxon:** taxonConceptID: Perlophiura profundissima; scientificName: Perlophiura profundissima; kingdom: Animalia; phylum: Echinodermata; class: Ophiuroidea; order: Ophiurida; family: Ophiuridae; genus: Perlophiura; scientificNameAuthorship: Belyaev & Litvinova, 1972; **Location:** waterBody: Pacific; stateProvince: Clarion Clipperton Zone; locality: UK Seabed Resources Ltd exploration claim UK-1; verbatimLocality: UK-1 Stratum A; maximumDepthInMeters: 4076; locationRemarks: RV Melville Cruise MV1313; decimalLatitude: 13.755833333333; decimalLongitude: -116.48666666667; geodeticDatum: WGS84; **Identification:** identifiedBy: Gordon Paterson, Adrian Glover, Helena Wiklund, Thomas Dahlgren; dateIdentified: 2015-06-01; identificationRemarks: identified by DNA and morphology; **Event:** samplingProtocol: Brenke Epibenthic Sledge; eventDate: 2013-10-17; eventTime: 01:50; habitat: Abyssal plain; fieldNumber: EB04; fieldNotes: Collected from epi net (on the epibenthic sledge); **Record Level:** language: en; institutionCode: NHMUK; collectionCode: ZOO; datasetName: ABYSSLINE; basisOfRecord: PreservedSpecimen

#### Description

**Figure 23.**
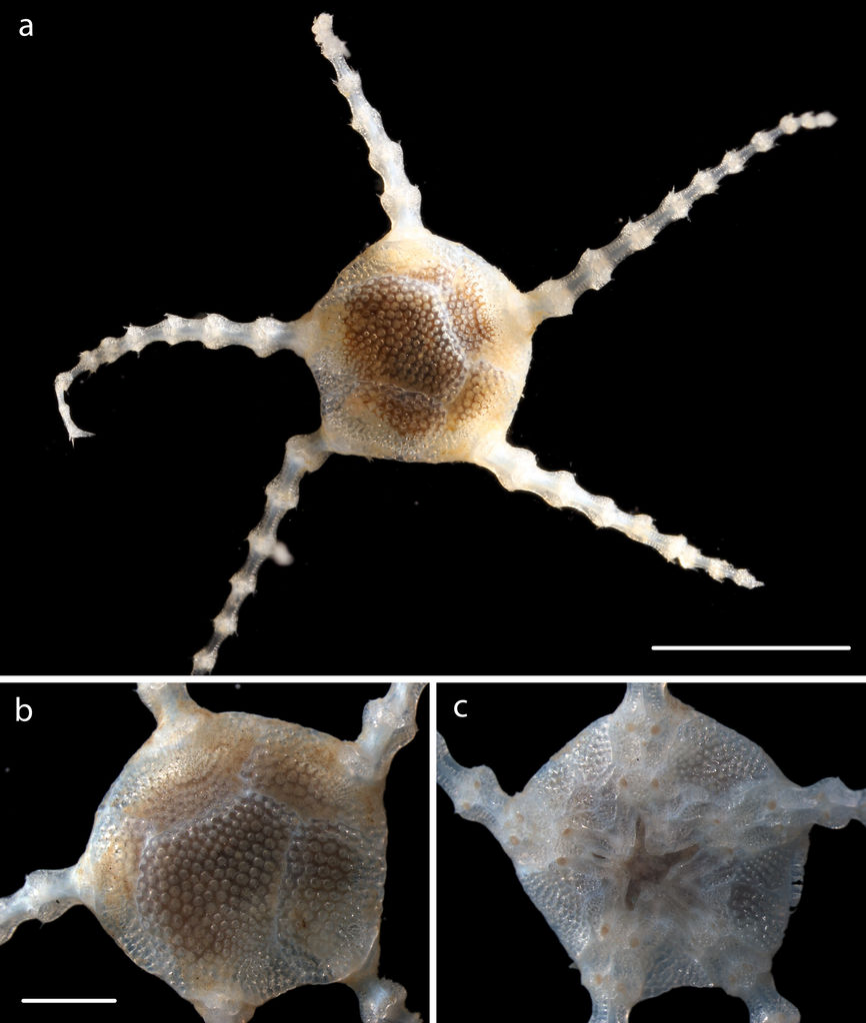
*Perlophiura profundissima* Belyaev and Litvinova, 1972. Specimen NHM_257. (a) Live specimen imaged dorsal side. (b). Dorsal surface detail. (c). Ventral surface detail. Scale bars (a) 3mm, (b) 1mm. Image attribution Glover, Dahlgren & Wiklund, 2015.

Specimen examined and matches *Perlophiura profundissima* Belyaev and Litvinova, 1972 (Belyaev and Litvinova 1972) voucher material, NHM_257, disc 3.1mm in diameter (Fig. 23).

Genetic data for this taxa with new GenBank accession numbers are provided in Table 2

#### Diagnosis

Forms a unique monophyletic clade distinct from other AB01 specimens. Morphologically agrees with *Perlophiura profundissima* but no genetic data available from type locality or any location for this taxon but type locality appears to be North Pacific at abyssal depths (Belyaev and Litvinova 1972).

## Discussion

Within the entire 6 million sq km Clarion Clipperton Zone, the best current online databases (OBIS 2015) list only 290 echinoderm records from 50 species. In this study, we report 48 new records from 17 species. This is an increase of ~25% for echinoderm species records from just a single 25-day cruise to a 30x30km location, with a relatively modest number of samples. All of our data are publically available through the Darwin Core outputs on this manuscript which are automatically fed into data aggregators such as GBIF and OBIS (Smith et al. 2013). All of our species determinations are supported by molecular DNA sequences, the data made available on GenBank and the voucher materials deposited in the Molecular Collections Facility of the Natural History Museum, London where they are available for future study by research visit or loan.

It is noteworthy that there was not a single 100% match for any of our sequences obtained with data on either NCBI GenBank or BOLD databases (BOLD 2015). This observation reinforces the point that there are very few taxonomic or genetic data available on the benthic biology of this region, an area undergoing intense exploration for mineral resources within the framework of the International Seabed Authority regulatory system (Wedding et al. 2015). The ISA has recently recognised the need for urgent action to make taxonomic data for the CCZ available from the large number of research cruises that are taking place supported by Sponsoring States (national governments) or private contractors (ISA 2014a). It is interesting to note that in the first 6 months of 2015 alone, there have been 3 large-scale benthic biology cruises to the eastern end of the CCZ, supported by the ABYSSLINE project (funded by UK Seabed Resources Ltd, cruise AB02), the German Government (the EU JPI cruise aboard the RV Sonne) and the EU 'MIDAS' programme (cruise JC120, partially funded by the Natural Environment Research Council, UK). As an example, from the ABYSSLINE cruise AB02 in March 2015 alone, we have an additional 289 echinoderm samples that are currently being identified and analysed for DNA; these results will be reported in future data papers over the course of the ABYSSLINE project.

The lack of comparative genetic data also has implications for our understanding of species ranges. We know that cryptic diversity is common in the deep sea (Knowlton 2000, Havermans et al. 2013, Jennings et al. 2013). With the routine use of molecular data in taxonomy, we will be better at detecting sibling species and defining species ranges, including for species considered to have distributions across multiple ocean basins and over very wide bathymetric ranges. To avoid exaggerated species ranges, we here follow a precautionary principle and have therefore avoided the use of taxon names based on morphological similarity alone unless the identity also is corroborated by a justifyable bathymetric and geographic proximity to type locality of the species name (i.e. abyssal Pacific).

The increased activity in terms of research cruises and sample collection in the CCZ make it more important than ever to provide taxonomic data quickly for an iterative building of baseline biodiversity knowledge in the CCZ region. Making these data available through rapid publication in open-access journals that support data-aggregator online systems is a key first step in this process.

## Supplementary Material

Supplementary material 1 Taxa table Data type: Genbank accession numbers
Brief description: List of taxa downloaded from GenBank that are included in the phylogenetic analyses, with their Genbank accession numbers. Accession numbers for taxa sequenced in this study are found in Table 2.
File: oo_73309.xlsx Glover et al.

Supplementary material 2 Tree file from Asteroidea phylogenetic analyses Data type: Phylogenetic
File: oo_61123.con Glover et al.

Supplementary material 3 Tree file from Crinoidea phylogenetic analyses Data type: Phylogenetic
File: oo_61124.con Glover et al.

Supplementary material 4 Tree file from Holothuroidea phylogenetic analyses Data type: Phylogenetic
File: oo_61125.con Glover et al.

Supplementary material 5 Tree file from Ophiuroidea phylogenetic analyses Data type: Phylogenetic
File: oo_61127.con Glover et al.

XML Treatment for Asteroidea sp. 'NHM_054'

XML Treatment for Freyastera benthophila

XML Treatment for Porcellanaster ceruleus

XML Treatment for Styracaster paucispinus

XML Treatment for Crinoidea sp. 'NHM_008'

XML Treatment for Crinoidea sp. 'NHM_055'

XML Treatment for Crinoidea sp. 'NHM_056'

XML Treatment for Crinoidea sp. 'NHM_300'

XML Treatment for Benthodytes sanguinolenta

XML Treatment for Psychropotes semperiana

XML Treatment for Amphioplus daleus

XML Treatment for Ophiomusium glabrum

XML Treatment for Ophiotholia sp. 'NHM_076'

XML Treatment for Ophiuroidea incertae sedis sp. 'NHM_041'

XML Treatment for Ophiuroidea incertae sedis sp. 'NHM_072'

XML Treatment for Ophiuroidea incertae sedis sp. 'NHM_303'

XML Treatment for Perlophiura profundissima

## Figures and Tables

**Table 1.** Primers used for PCR and sequencing of 18S, 16S and COI.

**Primer**	**Sequence 5'-3'**	**Reference**
18S		
18SA	AYCTGGTTGATCCTGCCAGT	Medlin et al. 1988
18SB	ACCTTGTTACGACTTTTACTTCCTC	Nygren and Sundberg 2003
620F	TAAAGYTGYTGCAGTTAAA	Nygren and Sundberg 2003
1324R	CGGCCATGCACCACC	Cohen et al. 1998
COI		
LCO1490	GGTCAACAAATCATAAAGATATTGG	Folmer et al. 1994
HCO2198	TAAACTTCAGGGTGACCAAAAAATCA	Folmer et al. 1994
polyLCO	GAYTATWTTCAACAAATCATAAAGATATTGG	Carr et al. 2011
polyHCO	TAMACTTCWGGGTGACCAAARAATCA	Carr et al. 2011
16S		
16SbrH	CCGGTCTGAACTCAGATCACGT	Palumbi 1996
Ann16SF	GCGGTATCCTGACCGTRCWAAGGTA	Sjölin et al. 2005

**Table 2.** Taxon treatments presented in this paper. Includes Natural History Museum global unique identifier (GUID) which link to the record in the Museum Data Portal (data.nhm.ac.uk), new NCBI GenBank accession numbers (GenBank#), ABYSSLINE Record number (ABYSSLINE_Record#) and NHM Molecular Collection Facility sample ID number (NHMUK_MCF#). Record numbers include both material archived at NHM (all tissue samples and DNA samples, plus some morphological material including whole specimens for some taxa) and material currently housed at the Craig R Smith lab, University of Hawaii (larger 'megafaunal' material indicated by CS numbers in the ABYSSLINE record#). GenBank numbers for data downloaded from GenBank for phylogenetic analysis are presented in Suppl. material 1.

**Class**	**Morphological identification**	**GUID**	**ABYSSLINE_** **record#**	**NHMUK_** **MCF#**	**Genbank_** **CO1#**	**Genbank_** **16S#**	**Genbank_** **18S#**
Asteroidea	Asteroidea sp. (NHM_054)	de4bd6ce-07fe-496e-bffc-67a4c6b9782c	NHM_054	185546311	---	KU519512	KU519530
Asteroidea	Asteroidea sp. (NHM_054)	bc03fc1a-3613-41a2-b1f1-bf905e0fa6d0	NHM_375	185546346	---	KU519528	---
Asteroidea	Freyastera cf. benthophila	b7ffe7a2-7be1-4d4f-b784-7aaecf0ee743	NHM_413 AB01_CS10	185546349	KU519550	KU519518	KU519535
Asteroidea	Freyastera cf. benthophila	16599946-2aba-4710-98e6-43c522061878	NHM_421	185546363	KU519551	---	---
Asteroidea	Porcellanaster cf. ceruleus	c57f1bd3-1b32-41e6-8e1d-0ad6472e4327	NHM_168	185546321	KU519568	---	---
Asteroidea	Porcellanaster cf. ceruleus	7e8ca2d8-aea1-45bd-b7e0-d0575cadd82d	NHM_200	185546325	KU519569	---	---
Asteroidea	Porcellanaster cf. ceruleus	95d0bd7f-0df9-47e4-8003-cd12007d54b4	NHM_253	185546332	KU519570	KU519525	KU519542
Asteroidea	Porcellanaster cf. ceruleus	d15a68e0-b2b3-40b4-8cab-0563609cc80d	NHM_267	185546329	KU519571	---	---
Asteroidea	Porcellanaster cf. ceruleus	76acc5a2-6e0e-4599-8104-b8e243af10c4	NHM_408	185546348	KU519572	---	---
Asteroidea	Styracaster paucispinus	4ae2430e-549e-47f2-ba5d-0e9a08443d31	NHM_374	185546345	KU519573	KU519527	KU519543
Crinoidea	Crinoidea sp. (NHM_008)	b2a871bf-46d5-4639-a839-427a3efa848c	NHM_008	185546315	KU519547	KU519514	KU519531
Crinoidea	Crinoidea sp. (NHM_055)	280c758b-5287-4a13-9f45-f6a6150b37d0	NHM_055	185546310	KU519548	KU519515	KU519532
Crinoidea	Crinoidea sp. (NHM_056)	92825c07-a16d-4c5e-a8e9-4fbcdc8cf44a	NHM_056	185546309	---	KU519516	KU519533
Crinoidea	Crinoidea sp. (NHM_300)	2866f91e-b99e-4703-a9d3-fe1876df1da1	NHM_300	185546328	KU519549	KU519517	KU519534
Holothuroidea	Benthodytes cf. sanguinolenta	d0062182-89dc-4deb-b746-688289783b5f	NHM_216 AB01_CS03	185546339	KU519546	KU519513	---
Holothuroidea	Psychropotes cf. semperiana	38c16bec-7bf9-4c2b-b862-5da460ba6c0c	NHM_220 AB01_CS05	185546335	---	KU519526	---
Ophiuroidea	Amphioplus cf. daleus	72db478a-ea4f-4f3e-be08-95ec9fb4d20e	NHM_094	185546316	KU519544	---	---
Ophiuroidea	Amphioplus cf. daleus	15e6ddc7-3ca7-453c-bba5-f84888716505	NHM_447 AB01_CS15	185546357	KU519545	KU519511	KU519529
Ophiuroidea	Ophiomusium cf. glabrum	c1c4d8f3-6cd5-439f-a546-943b5e2e8d8f	NHM_009	185546314	KU519552	---	---
Ophiuroidea	Ophiomusium cf. glabrum	4d6f6aaf-93fd-4629-b224-2ce8dd3320f6	NHM_124 AB01_CS02	185546319	KU519553	---	---
Ophiuroidea	Ophiomusium cf. glabrum	2ed865af-1605-4d78-8fd8-9c7659781854	NHM_256	185546331	KU519554	---	---
Ophiuroidea	Ophiomusium cf. glabrum	11948cb9-654f-4519-a654-f134380093ea	NHM_329 AB01_CS06	185546341	KU519555	KU519519	KU519536
Ophiuroidea	Ophiomusium cf. glabrum	292bd655-83d6-440f-9668-82dfa4185b04	NHM_335	185546342	KU519556	---	---
Ophiuroidea	Ophiomusium cf. glabrum	68072fc9-3e84-4202-8e97-6c9c0c5fc83d	NHM_415 AB01_CS12	185546351	KU519557	---	---
Ophiuroidea	Ophiomusium cf. glabrum	5ad996fe-134a-4625-a404-9d0cdae435d4	NHM_452	185546352	KU519558	---	---
Ophiuroidea	Ophiotholia sp. (NHM_076)	bd6fe2ce-b4ae-470e-8bdc-cf28a94c6208	NHM_076	185546306	KU519559	KU519520	KU519537
Ophiuroidea	Ophiotholia sp. (NHM_076)	97d40306-fe6c-4911-8e68-1f9efc3d838f	NHM_078	185546305	KU519560	---	---
Ophiuroidea	Ophiotholia sp. (NHM_076)	479218ae-813b-4736-b3f2-7eec63640ffd	NHM_104	185546317	KU519561	---	---
Ophiuroidea	Ophiotholia sp. (NHM_076)	90e22ace-ef5d-4cb5-a4a5-29fcd55ed660	NHM_119	185546318	KU519562	---	---
Ophiuroidea	Ophiuroidea incertae sedis sp. (NHM_041)	608349ff-5adf-4e1e-8cd7-7e0e41aee222	NHM_041	185546312	KU519563	KU519521	KU519538
Ophiuroidea	Ophiuroidea incertae sedis sp. (NHM_072)	241d094a-568f-4194-997c-fd08f67dcdac	NHM_072	185546307	KU519564	KU519522	KU519539
Ophiuroidea	Ophiuroidea incertae sedis sp. (NHM_303)	e9f38ce3-5ed5-49f3-8713-c26de2eefd2b	NHM_303	185546340	KU519565	KU519523	KU519540
Ophiuroidea	Ophiuroidea incertae sedis sp. (NHM_303)	93b0a70d-c74e-4735-b70e-0c6e4c6a36ff	NHM_371	185546344	KU519566	---	---
Ophiuroidea	*Perlophiura profundissima*	f263bc90-6307-462c-9e02-7b87d20e2840	NHM_257	185546330	KU519567	KU519524	KU519541
